# Comparative Life Cycle Transcriptomics Revises *Leishmania mexicana* Genome Annotation and Links a Chromosome Duplication with Parasitism of Vertebrates

**DOI:** 10.1371/journal.ppat.1005186

**Published:** 2015-10-09

**Authors:** Michael Fiebig, Steven Kelly, Eva Gluenz

**Affiliations:** 1 Sir William Dunn School of Pathology, University of Oxford, South Parks Road, Oxford, United Kingdom; 2 Department of Plant Sciences, University of Oxford, South Parks Road, Oxford, United Kingdom; Seattle Biomedical Research Institute, UNITED STATES

## Abstract

*Leishmania* spp. are protozoan parasites that have two principal life cycle stages: the motile promastigote forms that live in the alimentary tract of the sandfly and the amastigote forms, which are adapted to survive and replicate in the harsh conditions of the phagolysosome of mammalian macrophages. Here, we used Illumina sequencing of poly-A selected RNA to characterise and compare the transcriptomes of *L*. *mexicana* promastigotes, axenic amastigotes and intracellular amastigotes. These data allowed the production of the first transcriptome evidence-based annotation of gene models for this species, including genome-wide mapping of trans-splice sites and poly-A addition sites. The revised genome annotation encompassed 9,169 protein-coding genes including 936 novel genes as well as modifications to previously existing gene models. Comparative analysis of gene expression across promastigote and amastigote forms revealed that 3,832 genes are differentially expressed between promastigotes and intracellular amastigotes. A large proportion of genes that were downregulated during differentiation to amastigotes were associated with the function of the motile flagellum. In contrast, those genes that were upregulated included cell surface proteins, transporters, peptidases and many uncharacterized genes, including 293 of the 936 novel genes. Genome-wide distribution analysis of the differentially expressed genes revealed that the tetraploid chromosome 30 is highly enriched for genes that were upregulated in amastigotes, providing the first evidence of a link between this whole chromosome duplication event and adaptation to the vertebrate host in this group. Peptide evidence for 42 proteins encoded by novel transcripts supports the idea of an as yet uncharacterised set of small proteins in *Leishmania* spp. with possible implications for host-pathogen interactions.

## Author Summary

*Leishmania* are single-celled parasites that are transmitted between animal hosts by the bite of sand flies. Once inside their animal hosts they abandon their extracellular habit and invade cells of the immune system, called macrophages. This oscillation between hosts requires the parasite to be able to adapt to dramatically different environments. To help unravel the multitude of biochemical, ultrastructural and lifestyle differences that distinguish these specialised life cycle stages we characterised and contrasted the transcriptomes of insect and mammalian adapted forms. Using bioinformatic approaches we revised the genome annotation and discovered nearly 1,000 new genes that had not been described before. We found that over 3,000 genes change in their expression to facilitate the change in host environment including those involved in specifying cell shape, extracellular appearance and biochemistry. Furthermore we reveal that an ancient chromosome duplication shared by all *Leishmania* species may have contributed to the adaptation of these globally important parasites to parasitism of vertebrates.

## Introduction

Trypanosomatids, vector-borne protists of the order Kinetoplastida, infect humans, animals and plants and pose a heavy global burden on health and economic development [1,2]. The human pathogenic *Trypanosoma brucei*, *T*. *cruzi* and *Leishmania* spp. affect mostly people in developing countries and together account for 4.4M disability-adjusted life years [3]. Infections with *Leishmania* spp. present as a spectrum of diseases ranging from cutaneous lesions to fatal visceral infections [4], estimated to cause 20,000 to 40,000 deaths per year [5]. Uniquely among trypanosomatids, *Leishmania* spp. are adapted to survival and replication in the phagolysosome of professional phagocytes, a niche in which only few pathogens of any lineage thrive. The shared biology and unique pathogenicity mechanisms of *Leishmania* spp. and trypanosomes have been the focus of intense research [1] and the genetic basis for the species-specific differences in disease manifestations remain key questions in post-genome analyses of these parasites.

Sequencing of over 20 trypanosomatid spp. genomes to date has revealed an extreme degree of synteny and high proportion of shared genes. Of the ~8,000 annotated genes in *Leishmania* spp. genomes, ~6,000 are shared with other trypanosomatids and 95% of genes are conserved between *L*. *major*, *L*. *infantum*, *L*. *braziliensis* and *L*. *mexicana*. Only ~200 to 400 genes were found to be absent from one or more of these genomes and surprisingly few genes are unique to any one *Leishmania* species [6,7]. Instead, heterogeneity has arisen through large-scale variation in gene and chromosome copy number [7] with widespread aneuploidy in natural *Leishmania* populations [8,9]. One event that is shared by all examined *Leishmania* spp. is a duplication of chromosome 31 (in *L*. *mexicana*, a fusion event joined chromosomes 8 and 29 and as a result the homologue of chromosome 31 is called “chromosome 30”). It is unknown what role this duplication event may have played in the evolution of the parasite. To pinpoint genetic adaptations that allowed *Leishmania* spp. to parasitise mammalian macrophages requires better knowledge about gene expression patterns specific to the intracellular amastigote forms.

Amastigotes are formed when metacyclic promastigote forms are egested by an infected sandfly during a bloodmeal and phagocytosed by a professional phagocyte. During the subsequent differentiation, the morphology of the parasite’s cell body changes from an elongated to an ovoid shape and the cells lose their motility; only the tip of their short flagellum remains external to the flagellar pocket and the flagellar axoneme is devoid of the molecular motors and accessory structures required for beating the flagellum [10]. The properties of the cell surface change: the promastigote lipophosphoglycan (LPG) coat is lost and amastin surface proteins are upregulated [11]. A change in metabolism shifts the cells from using glucose and proline as their carbon source to beta-oxidation of fatty acids and increased use of amino acids [12,13]. Known virulence factors expressed in amastigote forms include superoxide dismutases, which protect against reactive oxygen species produced by the host cell [14], the major surface protease gp63 (also known as leishmanolysin) [15], cysteine peptidases [16], the iron transporter LIT1 [17] and A2 proteins, which were linked to the establishment of visceral infections [18].

To study amastigote biology, parasites have been isolated from infected animals or *in vitro* infected macrophages or alternatively, generated from promastigotes in cell-free medium through a decrease of pH and increase in temperature (“axenic amastigotes”). The latter show the characteristic amastigote morphology and exhibit many of the molecular and biochemical characteristics of lesion-derived amastigotes [19–21] but their virulence was shown to be attenuated compared to lesion-derived amastigotes [22]. Thus, whilst they offer the opportunity to study amastigote-specific molecular processes in a system that yields much higher numbers of cells than purification of amastigotes from macrophages and free of contaminating host cell material, it remains controversial how representative their biology is of “true” amastigotes.

Microarray-based studies comparing gene expression profiles of promastigotes and amastigotes (generated axenically, or isolated from tissue culture macrophages or lesions) found that most transcripts were constitutively expressed but each study identified a few genes that showed strong stage-regulated mRNA expression, including genes affecting morphology, translation and amastigote-specific virulence factors [23–26], with notable differences between *L*. *infantum* axenic and intracellular amastigotes [27]. Combined proteomic and transcriptomic studies comparing axenic amastigotes to promastigotes found overall modest correlations between mRNA and protein levels [28]. A time-course analysis of differentiation revealed that early during differentiation changes in RNA levels were pronounced while at later time points downregulation of translation dominated [29].

RNA-sequencing (RNA-seq) technology now allows discovery of new information about the transcriptomes of *Leishmania* [30–32]: it yields measurements of relative transcript abundance over a larger dynamic range (capturing most of the genes in the genome) and identifies the precise nucleotide sequence of transcripts including transcript boundaries. This is particularly important in *Leishmania* spp., where regulation of gene expression occurs post-transcriptionally and sequences in 5’ and 3’ UTRs have been shown to mediate differential transcript abundances and translation between life cycle stages [33–37]. Moreover, RNA-seq analyses readily uncover novel transcripts and have facilitated refinement of genome annotations of a variety of species ranging from bacteria to metazoa [38–41] including *T*. *brucei* procyclic forms [42] and *L*. *major* promastigote forms [30]. Finally, RNA from intracellular pathogens can be sequenced together with host cell RNA (“dual RNA-seq” [43,44]), eliminating the need for cell purification procedures that might affect gene expression patterns prior to RNA extraction.

Here we used RNA-sequencing of *L*. *mexicana* to profile the transcriptomes of promastigotes and early amastigotes, 24 hours after exposure to differentiation conditions, when morphological transformation is complete and well-characterised molecular markers of amastigotes are upregulated. The amastigotes were derived from the same population of promastigotes either by infection of bone marrow derived murine macrophages or differentiation in axenic culture, allowing a comparison of gene expression patterns of intracellular and axenic amastigotes with a known history and at a defined stage in development.

We utilised the RNA-seq data to define precisely the genomic positions of spliced leader acceptor sites and poly-A addition sites and used this information to refine the current set of gene model predictions for *L*. *mexicana*. Here we found evidence for extensions and truncations of annotated coding sequences and 936 novel transcripts. Using this novel RNA-seq guided annotation of 9,169 predicted coding sequences, we quantified transcript abundances and tested for differential expression between life cycle stages. We found that 41% of all genes showed statistically significant changes in relative mRNA abundance between promastigotes and intracellular amastigotes and 13% between axenic and intracellular amastigotes. Whilst this showed that axenic differentiation did not fully replicate the intracellular development of amastigotes, less than 1% of all transcripts varied more than two-fold between the two amastigote forms, pointing to a fundamentally similar pattern of gene expression. Over one third of amastigote enriched transcripts encode novel and hypothetical proteins, many conserved only within *Leishmania* spp. Furthermore, genes upregulated in amastigotes are significantly enriched on chromosome 30, suggesting that amastigote-specific functions may be a driving force in maintaining supernumerary copies of this chromosome.

## Results

### Transcriptome sequencing of promastigotes, axenic amastigotes and intracellular amastigotes

#### Isolation of RNA

**Fig 1:**
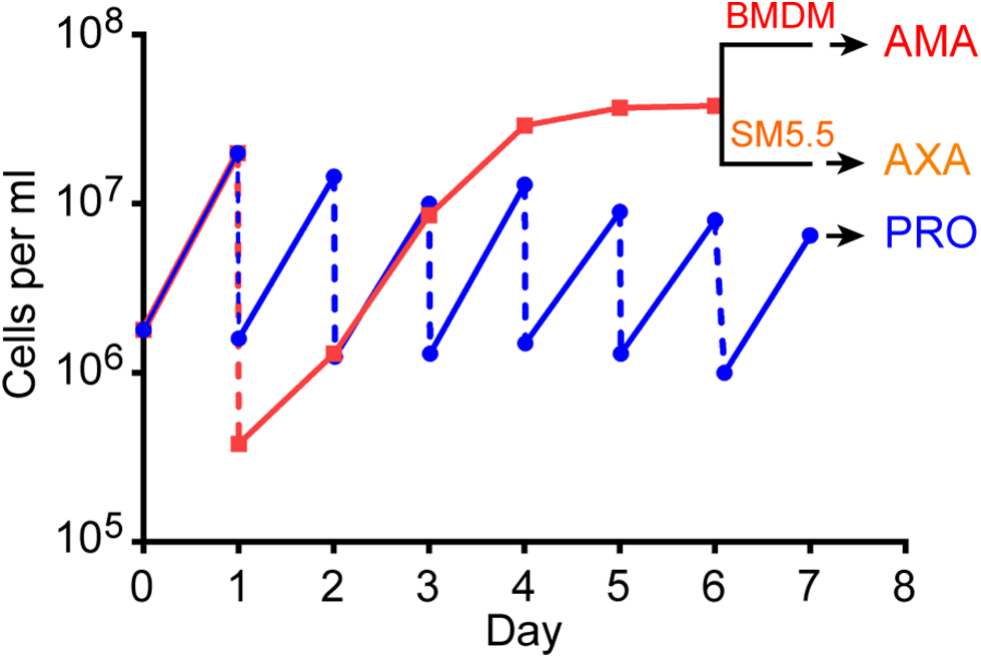
Growth history of cells used for RNA extraction. Data shows growth curve for one of the three replicates. *L*. *mexicana* promastigotes (PRO) were maintained in exponential growth by diluting the culture to 1x10^6^ cells ml^-1^ every day (blue line). A second promastigote culture was inoculated with 1–2.5x10^5^ cells ml^-1^ and left to grow for five days to stationary phase (red line). Stationary phase promastigotes were used to infect bone marrow derived macrophages (BMDM) to produce intracellular amastigotes (AMA) or differentiated to axenic amastigotes (AXA) in Schneider’s medium (SM5.5) for 24h. RNA was extracted from AMA, AXA and PRO on day 7.

To profile and compare gene expression patterns of promastigotes (PRO), axenic amastigotes (AXA) and intracellular amastigotes (AMA) we took advantage of the culture systems that allow production of these *L*. *mexicana* cell forms *in vitro* [45]. Total promastigote RNA was extracted from three separate populations in the exponential phase of growth (Fig 1). To generate amastigotes, stationary phase promastigotes were either passaged into Schneider’s Drosophila medium at pH5.5 [20] for the generation of AXA or used for infection of murine bone marrow derived macrophages (BMDMs) to produce AMA. Total RNA was extracted from AXA and AMA 24h later (Fig 1). A sample from the infected BMDM populations was set aside at the time of RNA isolation for microscopic examination and quantification of infection levels. The percentage of infected BMDMs in the three replicates ranged from 70.4% to 94.6% with an average ratio of 5.3 parasites per macrophage (S1 Fig).

To determine whether RNA could be recovered quantitatively from both mouse and *L*. *mexicana* in the infected BMDM population, we compared the parasite load established from microscopic examination of the infected BMDMs (S1 Fig) with the relative amounts of leishmanial and murine rRNA in the three AMA samples. In kineotplastids, the LSU rRNA is fragmented into multiple molecules: two large ones (LSU α and LSU β) and four small ones, which can be separated from the murine 28S rRNA by gel electrophoresis (S2 Fig). The ratio of leishmanial LSU β RNA to murine 28S RNA calculated from electropherograms correlated well with the parasite loads in each sample (R^2^ = 0.90; Table 1).

**Table 1:** Estimate of *L*. *mexicana* RNA in mixed RNA samples from infected BMDMs.

Sample	AMA1	AMA2	AMA3
Amastigotes per 100 macrophages	207	636	748
LSU β (1.6 kb) peak size in AFU	3.4	4.8	8.4
28S (4.7 kb) peak size in AFU	36.6	16.5	17.5
Relative *L*. *mexicana* rRNA amount	2.1	3.0	5.3
Relative murine rRNA amount	7.8	3.5	3.7
Estimated proportion of *L*. *mexicana* RNA in sample	21.2%	46.1%	58.9%
Percentage of reads mapping to *L*. *mexicana* genome	25.6%	54.9%	61.2%

Peak sizes of the LSU β and 28S rRNA subunits were measured in arbitrary fluorescence units (AFU) on an Agilent Bioanalyzer. To estimate the proportion of *L*. *mexicana* RNA in the sample, AFU were divided by length of the corresponding rRNA to determine the relative amounts of leishmanial and murine rRNAs. The number of amastigotes per 100 macrophages was determined by light microscopy.

#### Sequencing

RNA samples were enriched for poly-adenylated messenger RNA by poly(dT)-selection and paired-end sequencing performed on an Illumina platform (see materials and methods). Paired end reads were mapped to a hybrid mouse-*L*. *mexicana* genome (Table 2). The proportion of reads from the three AMA samples that mapped to the mouse and *L*. *mexicana* genomes, respectively, correlated well with parasite load (R^2^ = 1) and with the relative rRNA amounts from each species (R^2^ = 0.97; Table 1).

**Table 2:** RNA-seq read mapping summary.

Sample	PRO1	PRO2	PRO3	AXA1	AXA2	AXA3	AMA1	AMA2	AMA3
**Random primed library**									
Total paired-end reads	12,835,963	13,087,624	12,858,646	13,124,114	13,748,422	12,778,984	13,477,640	13,357,246	13,044,810
Low-quality reads	20,538	23,558	29,575	17,061	34,371	17,891	9,434	13,357	11,740
Reads mapping to multiple loci	4,133,727	3,572,451	4,109,315	5,155,858	4,552,037	4,044,110	1,187,608	2,910,706	3,115,683
Genes with non zero read counts	9,129	9,119	9,119	9,133	9,129	9,125	9,112	9,123	9,123
Total reads mapped to *L*. *mexicana* genome	12,220,790	12,301,132	12,109,395	12,525,089	12,950,160	11,893,323	3,437,982	7,224,190	7,767,651
Reads with no match in genome	594,635	762,934	719,676	581,964	763,891	867,770	10,030,224	6,119,699	5,265,419
Total reads with SL sequence (last 12nt)	673,297	1,067,867	937,353	786,722	1,009,393	1,225,884	262,651	499,028	572,112
Mapped reads with SL sequence (last 12nt)	665,046	1,051,560	923,254	778,432	994,815	1,208,732	259,961	493,948	566,435
**T15VN PRIMED LIBRARY**									
Total reads	nd	25,956,366	22,854,437	nd	21,935,789	20,450,384	nd	23,956,594	25,696,946
Low-quality reads	nd	8,012,973	8,617,839	nd	6,910,070	5,436,250	nd	9,613,243	8,996,465
Total reads with poly(A) tail (≥5 A)	nd	2,097,561	2,088,542	nd	2,200,926	2,149,944	nd	1,793,186	1,476,286
Mapped reads with poly(A) tail (≥5 A)	nd	756,459	767,004	nd	805,680	812,005	nd	424,491	373,912

### Genome-wide mapping of spliced-leader acceptor and polyadenylation sites

To map the 5’ ends of transcripts, defined by the position of spliced-leader acceptor sites (SLAS), reads generated from the random primed library that contained the spliced leader sequence (SL) were mapped to the *L*. *mexicana* genome (see materials and methods and [46]). In total, 6,942,183 SL-containing reads were mapped to 21,249 positions in the genome (S1 Table). Ninety-six percent of SLAS mapped to an AG dinucleotide consistent with the known conservation of this dinucleotide at the vast majority of mapped kinetoplastid trans-splice sites [30,42]. To map the sites at which poly-A tails were added to transcript 3’ ends (PAS) a second T15VN-primed library was generated from two of the RNA samples for each cell type (see materials and methods). 3,939,551 reads containing at least 5 consecutive A nucleotides at the 3’ end were mapped to 96,522 positions in the genome (materials and methods, [46]) (S2 Table).

### Gene models based on RNA-seq data predict 936 novel genes

Assignment of SLAS and PAS to genes was initially performed using version 6.0 of the *L*. *mexicana* genome. We found that in the majority of cases (6,796 annotated protein coding genes) there was good correspondence between an annotated coding sequence (CDS), RNA-seq read coverage and positions of SLAS and PAS. We did however find many loci bounded by SLAS at the 5’ end PAS at the 3’ end suggestive of processed transcripts from genes that had not been annotated. Many of these putative novel transcripts contained open reading frames that could represent unannotated CDS. This prompted us to define gene models for predicted protein coding genes guided by the RNA-sequencing data (for details of gene predictions see materials and methods).

**Fig 2:**
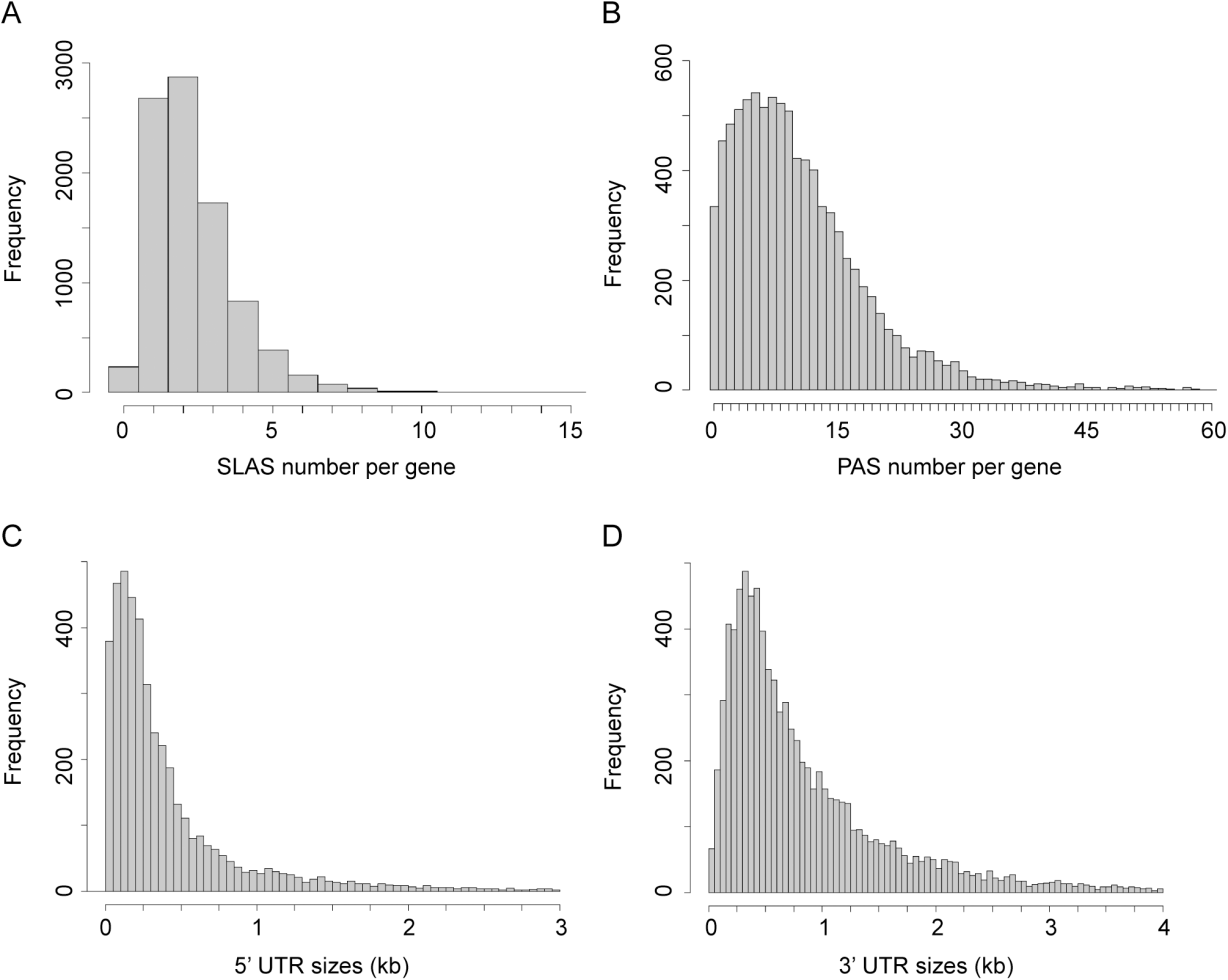
Distribution of SLAS, PAS and UTR lengths. (A) Distribution of assigned SLAS numbers per gene. A SLAS was assigned if at least nine SL-containing reads were mapped to this position across all nine random primed libraries (n = 20,812). (B) Distribution of assigned PAS numbers per gene (n = 95,097). A PAS was assigned if at least six reads terminating in ≥ 5 A were mapped to this position across all six T15VN primed libraries. (C) Distribution of 5’ UTR lengths (without the SL sequence; n = 9,029). (D) Distribution of 3’ UTR lengths (n = 9,029).

In total our combined analysis of the transcriptome of PRO, AMA and AXA and the existing annotation of the *L*. *mexicana* genome predicted a total of 9,169 putative protein coding genes, of which 936 have not been previously described. A SLAS could be assigned to 8,882 genes and a PAS to 8,769 genes; for 8,540 genes both a SLAS and PAS were assigned and only 58 genes had neither. The position of the SLAS indicated that 1,253 genes had an upstream ATG start codon in-frame with the annotated CDS (‘extended CDS’ (S3 Table)) and for 184 genes the SLAS was mapped to a position downstream of the annotated ATG (‘truncated CDS’ (S4 Table)). The majority of genes had between 1 and 3 SLAS, with a mean of 2.4 (Fig 2A). For 8,045 transcripts (90.6%) the SLAS with the most counts was the same in PRO and AMA. The mapped 3’ ends of transcripts showed greater heterogeneity than the 5’ ends, with a mean of 10.9 PAS per gene (Fig 2B). The median lengths of the untranslated regions (UTRs), based on the gene models defined above, was 242 nt for 5’ UTRs (Fig 2C) and 584 nt for 3’ UTRs (Fig 2D). On average, UTRs and intergenic regions are longer in *Leishmania mexicana* than in *T*. *brucei* [42,47]. There was no correlation between 5’ UTR and 3’ UTR length on the same gene, or between the length of a UTR and the abundance of that mRNA within the cell (S3 Fig).

### Characterisation of the predicted novel genes

#### A subset of novel transcript sequences are conserved in multiple kinetoplastid species

**Fig 3:**
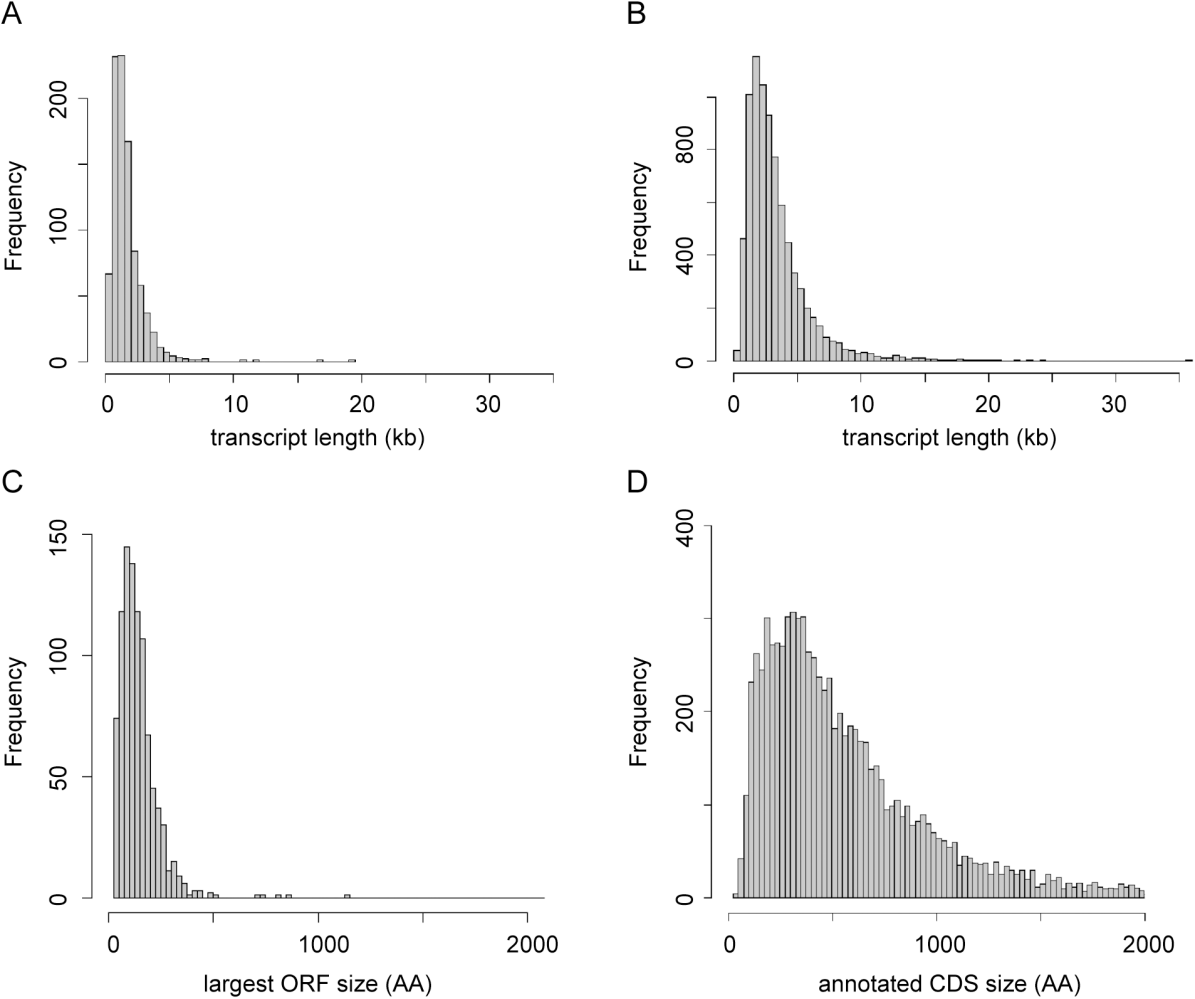
Characterisation of novel transcripts. (A) Size distribution of novel transcripts (n = 936). (B) Size distribution of transcripts derived from genes annotated in TriTrypDB V6 (n = 8,250). (C) Size distribution of largest ORFs found on the sense strand of novel transcripts (n = 936). (D) Size distribution of CDS annotated in TriTrypDB V6 (n = 8,250).

**Fig 4:**
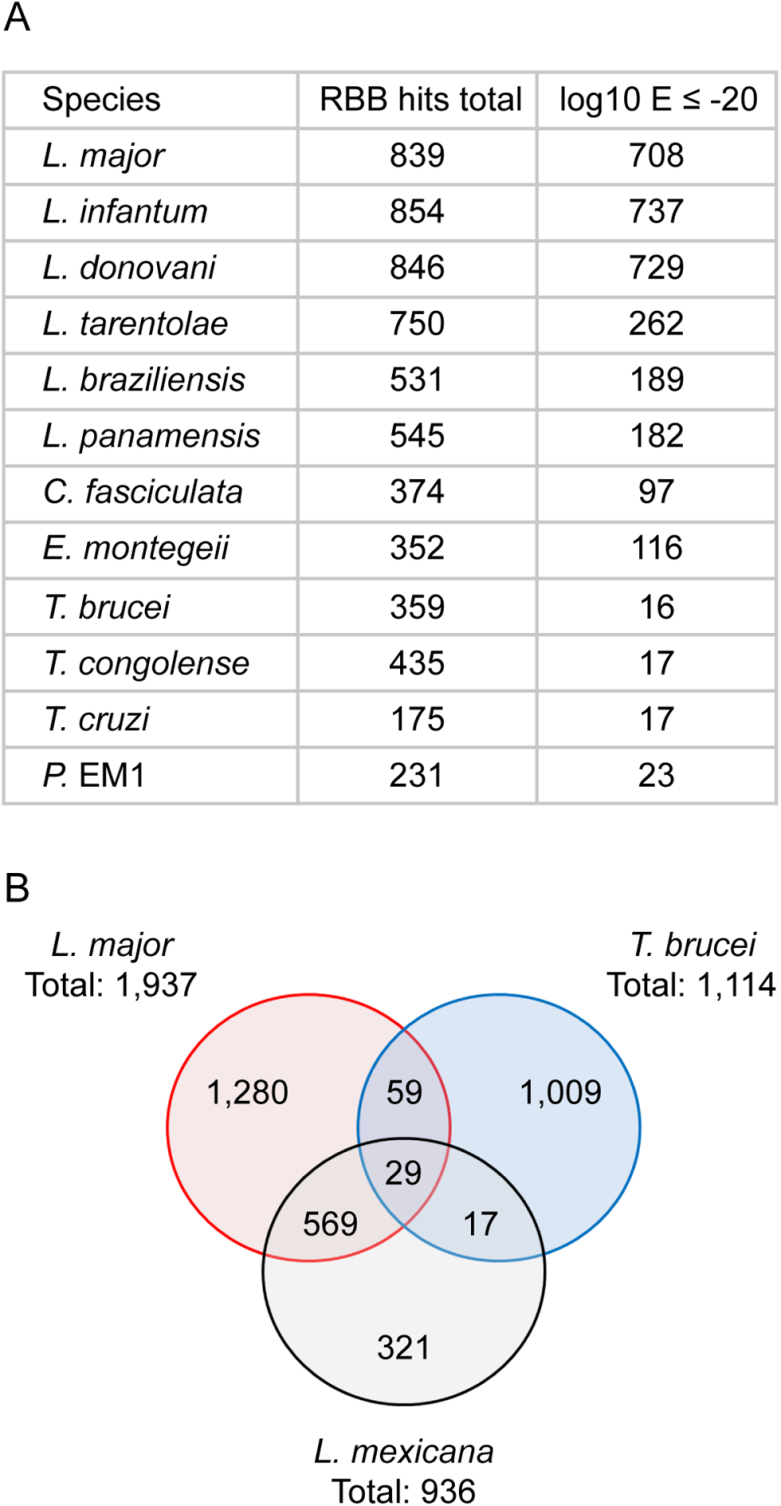
Conservation of novel transcript sequences. (A) The novel transcripts were used as query sequences in a reciprocal best tblastx search of 12 kinetoplastid genomes. “RBB hits total” indicates the number of reciprocal best tblastx hits returned; “log10 E ≤ -20” indicates the number of hits returned with an E value ≤ 10^−20^ for the reciprocal tblastx search. (B) Venn diagram showing the number of hits returned in a series of reciprocal best tblastx searches comparing the novel transcripts found in *L*. *mexicana* (this study), *L*. *major* [30] and *T*. *brucei* [42].

**Fig 5:**
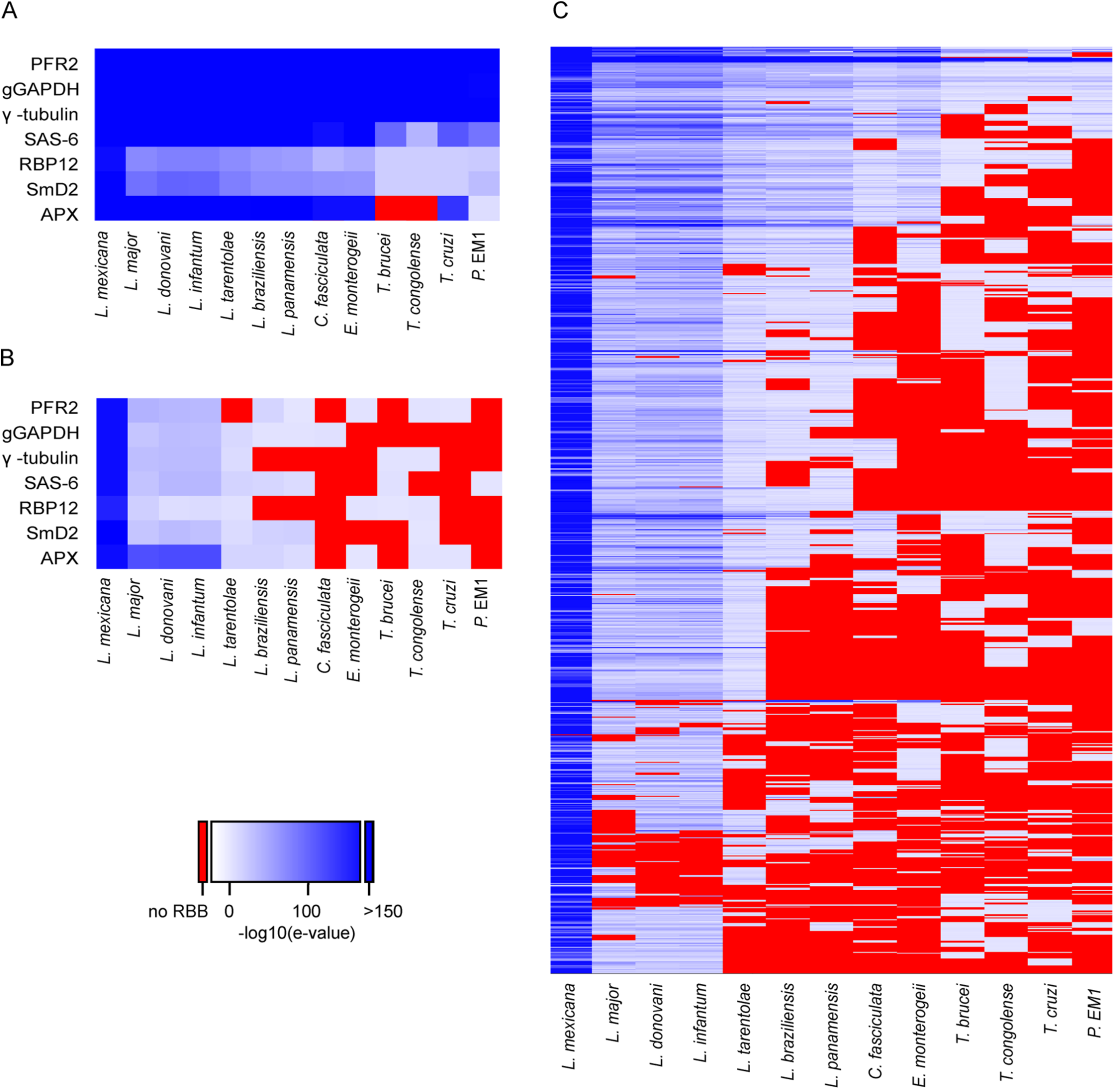
Conservation of novel transcript sequences across kinetoplastid genomes. The 936 novel *L*. *mexicana* transcripts and 7 control genes were used as queries in tblastx searches of 12 kinetoplastid genomes and the best hits were then used in a reciprocal tblastx search against the complete *L*. *mexicana* genome. The heat maps indicate the E value of the returning hits, with darker shades of blue representing lower E values. Sequences that did not return a hit are represented in red. (A) Sequences used as positive controls for conserved CDS (Gene IDs: PFR2, LmxM.16.1430; gGAPDH, LmxM.29.2980; γ-tubulin, LmxM.25.0960; SAS-6, LmxM.34.4280; RPB12, LmxM.20.0490; SmD2, LmxM.32.3190; APX, LmxM.33.0070). (B) Intergenic sequences downstream of the CDS in (A), used as negative controls. (C) Each row represents one of the 936 novel *L*. *mexicana* transcripts.

The pattern of SLAS and PAS indicated there were 936 putative novel mRNA transcripts (S5 Table), which were shorter, on average, than the transcripts for the annotated genes (mean length of 1,656 nt (Fig 3A) compared with 3,298 nt (Fig 3B) for the annotated genes). Consequently, ORFs identified in the novel transcript sequences (mean 80 codons, median 58 codons) were significantly shorter than the CDS of annotated protein coding genes (mean 628 codons, median 465 codons; KS test, p < 2.2x10^-16^) (Fig 3C and 3D). To identify ORFs within the novel transcript sequences that showed protein sequence conservation in other species, the novel transcripts were used as query sequences in tblastx analyses of 12 kinetoplastid genomes. The number of returned reciprocal best tblastx hits ranged from 175 in *T*. *cruzi* to 854 in *L*. *infantum* (Fig 4A and S6 Table). To validate this method, the same reciprocal best tblastx analysis was performed on a control set of 7 genes encoding widely conserved proteins (the paraflagellar rod protein PFR2, γ-tubulin, the basal-body protein SAS-6, RNA polymerase II subunit RPB12, small nuclear ribonucleoprotein SmD2, glycosomal glyceraldehyde 3-phosphate dehydrogenase (GAPDH) and ascorbate peroxidase (APX)). This test showed that this method robustly identified the conservation patterns for these genes (Fig 5A).

A random 1kb sequence with a GC content of 59.7% (*L*. *mexicana* genome average) is expected to contain on average 5.4 ORFs of at least 25 codons. Spurious ORFs that occur by chance in *L*. *mexicana* intergenic sequences could result in tblastx matches with the homologous intergenic sequences of its closest relatives and thus potentially create false positive assignment of coding sequences. To demonstrate that these novel genes were not just spurious ORFs a control BLAST search was performed. Here the intergenic sequences downstream of the 7 control genes were used as queries. As expected, high GC content intergenic sequences that are not thought to encode proteins can produce reciprocal best tblastx hits with very low Expect (E) values in closely related species (the *Leishmania Leishmania* spp. *L*. *major*, *L*. *infantum*, *L*. *donovani*) (Fig 5B). However, in more distantly related *Leishmania Viannia* spp. (*L*. *braziliensis*, *L*. *panamensis*), the control set of intergenic sequences did not return any reciprocal best tblastx E values < 10^−18^ and no E value < 10^−5^ in *Crithidia*, *Trypanosoma* spp. and *Phytomonas*. We therefore used the E values returned in the search with intergenic sequences to set a cut-off to identify those novel transcript sequences that returned E values lower than any of the intergenic control sequences. About one in five of the novel transcripts returned reciprocal tblastx hits with E values ≤10^−20^ across *Leishmania Leishmania and Leishmania Viannia* spp., but not in *Trypanosoma* spp.; these may represent *bona fide* conserved protein coding sequences specific to *Leishmania* spp. 19 novel transcript sequences returned strong reciprocal best tblastx hits with E values ≤10^−20^ from *Leishmania spp*., *Trypanosoma* spp. and *Phytomonas* genomes (Figs 4A and 5C and S6 Table) indicating that the coding potential of these sequences is conserved in these genera. A separate reciprocal tblastx analysis comparing the 936 novel *L*. *mexicana* transcripts with the novel transcripts reported for the promastigote form of *L*. *major* [30] found 598 hits. 46 hits (7 with E values ≤10^−20^) were returned in a comparison with the 1,114 novel transcripts identified in procyclic *T*. *brucei* [42] and 88 were identified in the comparison between *T*. *brucei* and *L*. *major* (Fig 4B). Taken together these results indicate that the majority of the 936 novel *L*. *mexicana* transcript sequences are conserved within *Leishmania* spp. with high conservation of predicted amino acid sequences in a subset of ~200 novel transcripts.

#### Mass spectrometry evidence for 47 of the novel proteins and support for predicted protein extensions

To find evidence that some of the novel transcripts are translated into proteins we performed mass spectrometry (MS) analysis of promastigote and axenic amastigote cell lysates (see materials and methods) and searched the resulting spectra against a custom database containing all annotated *L*. *mexicana* protein sequences plus a three-frame translation of the 936 novel transcripts. We also analysed a published dataset of proteins from intracellular amastigotes [48] (materials and methods). In total, we found unique peptide matches for 47 of the novel transcript sequences and 3,835 proteins that had already been annotated; 42 novel proteins were only identified in the PRO and AXA samples (S7 Table), 5 only in the AMA dataset (S8 Table) and 15 in both. A scan for Pfam domains identified a Pfam-A domain in five of the 47 novel proteins identified by MS (two histones, one Ribosomal_S27e, one Thioredoxin and one zf-RanBP domain). A further 30 novel transcript sequences that had no peptide match in our MS data also produced significant Pfam-A domain hits (S9 Table), which supports the prediction that these are protein coding genes. A third (14) of the 47 novel proteins identified in our MS data had reciprocal best blast hits among the novel *T*. *brucei* transcripts [42] and for 12 of these, Ericson et al. [49] recently reported independent MS evidence.

The *L*. *mexicana* MS data was also queried to find evidence for the predicted 5’ extensions to CDS. Of the predicted 1,253 extended proteins, 433 were identified by MS (i.e. peptides were found that mapped to any part of the predicted protein sequence) and 116 of these had unique peptides mapping to the predicted N-terminal extensions (S10 Table) confirming the RNA-seq guided gene model.

#### uORFs

The transcripts for the annotated CDS were examined for the presence of ORFs (≥25 codons) upstream of the CDS (uORFs). uORFs were found in 1,122 transcripts (S11 Table) but MS did not identify any peptides mapping to these uORFs.

### Transcriptome profiling

We next analysed transcript abundances in each of the three *L*. *mexicana* cell forms by calculating the number of fragments per kilobase of transcript per million mapped reads (FPKM) for each sample (S12 Table and S4A Fig). The correlation between biological replicates was between 0.90 and 0.99 (R^2^ (Pearson), log_10_ FPKM values) (S13 Table) with low coefficient of variation (S4B Fig). These results demonstrate high levels of agreement and low amounts of variability across the range of expression levels observed in each of the biological replicates. The three AMA samples showed lower FPKM values than the AXA and PRO samples because the AMA reads were derived from a mixed library of leishmanial and murine RNA.

**Fig 6:**
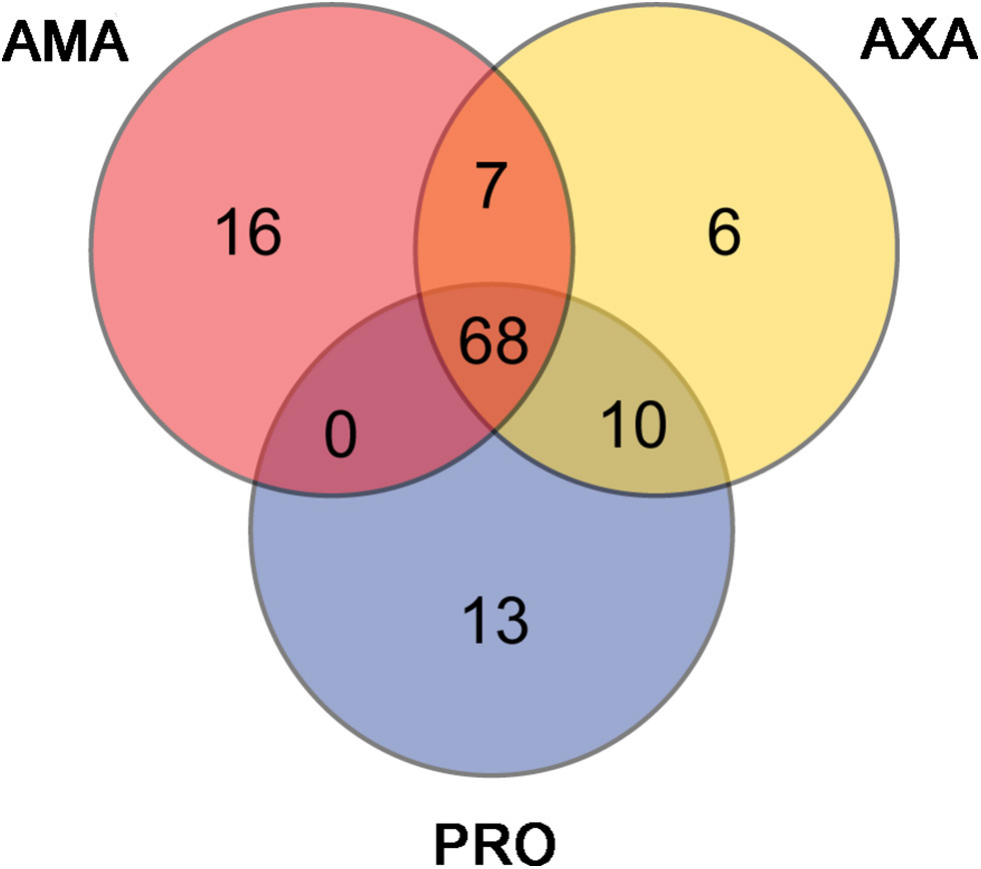
The majority of highly expressed transcripts are shared between AMA, AXA and PRO. The Venn diagram shows for each cell type the 91 transcripts comprising the top percentile of FPKM values and indicates the extent of overlap between the three datasets.

We examined the genes comprising the top FPKM percentile in each cell form (91 genes; (S14 Table)) to discover the most abundant transcripts in each condition and to assess the extent of overlap. Sixty-eight genes (75%) were shared between the top FPKM percentile in PRO, AXA and AMA (Fig 6 and S14 Table), including 45 encoding ribosomal proteins (42 in reference annotation, 4 encoded by novel transcripts [LmxM.18_241026, LmxM.24_804446, LmxM.24_805244, LmxM.24_806159]), 10 histones (9 in reference annotation, 1 novel [LmxM.21_369741]), 2 heat shock proteins and 2 novel proteins of unknown function (no Pfam domains [LmxM.20_617046, LmxM.32_1186260]). Thirty-three genes (25%) were only in the top FPKM percentile in one of the cell forms (13 in PRO, 6 in AXA and 16 in AMA). The latter included genes encoding 3 cysteine peptidases (known amastigote virulence factors), amastin, 2 hypothetical and 2 novel proteins (LmxM.19_375604, LmxM.33_1093342). Closer inspection of all thirteen novel transcripts in the top FPKM percentiles showed that one (LmxM.16_570431) corresponded to the 3’ UTR of PFR2, indicating that collapsed gene arrays in the genome assembly could cause false positives in the annotation of novel transcripts. Conversely, MS evidence proved the existence of a protein product from three of the remaining twelve, including LmxM.19_375604, which subsequent analysis (next section) showed to be significantly upregulated in AMA compared to PRO.

### Over 3,000 genes are differentially expressed between PRO and AMA

**Fig 7:**
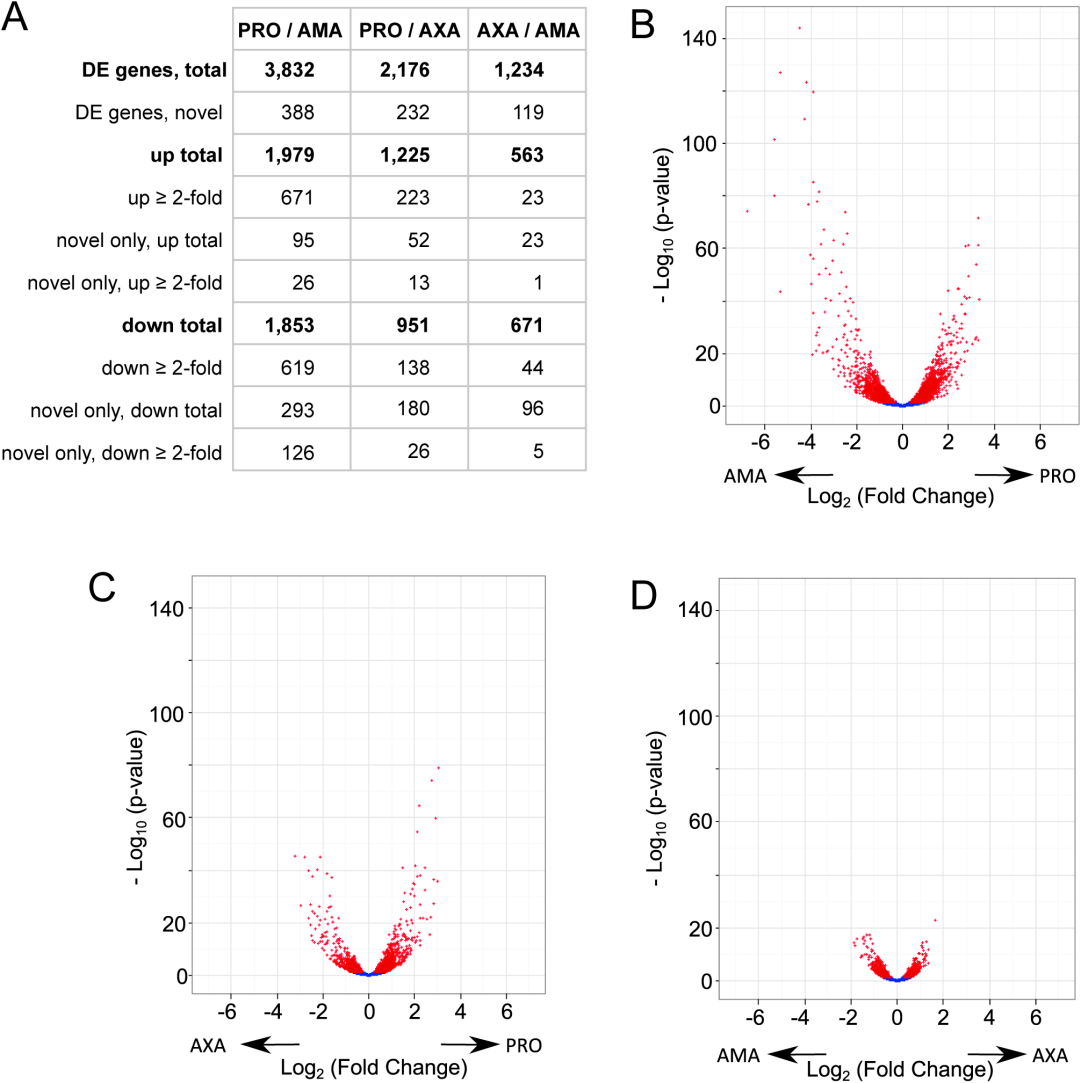
Differential gene expression between AMA, AXA and PRO. (A) Table summarising the number of differentially expressed (DE) genes in each pair-wise comparison; “novel” refers to the 936 novel transcripts defined in this study. (B-D) Volcano plots for the comparisons between PRO and AMA (B), PRO and AXA (C), AXA and AMA (D). Each dots represents one transcript; red denotes differential expression (padj ≤ 0.05). Arrows indicate the cell type showing higher expression.

To identify the genes that were differentially expressed we performed pairwise differential expression testing between all three cell forms. The results showed that there was a significant (Benjamini-Hochberg corrected p-value ≤ 0.05) difference between PRO and AMA in the abundance of 3,832 transcripts (388 of these represent novel genes and 1,290 showed at least a 2-fold change) (Fig 7A and S15 Table), 2,176 transcripts differed in abundance between PRO and AXA (232 novel genes; 361 with ≥ 2-fold change) (Fig 7B and S16 Table) and 1,234 transcripts differed in abundance between AMA and AXA (119 novel genes; 67 genes with ≥ 2-fold change) (Fig 7C and S17 Table).

We validated the differential expression data by analysis of 13 genes where published Northern blot data was available comparing *L*. *mexicana* RNA abundance in PRO with AMA or AXA (S18 Table): five genes linked to the *PFR2* locus [50], three glucose transporter genes, *LmGT1*, *LmGT2* and *LmGT3* [51] and five other genes. For 9 of the 13 genes the differential expression analysis fully agreed with the published data. For three genes, where different expression levels had been detected by Northern blot, our analysis found no significant difference (LmxM.16.0390, LmxM.16.1410 and LmxM.16.1410). For *LmGT3*, reported to be expressed at similar levels in PRO and AXA [51], the RNA-seq data showed a small but significant increase in AXA. Interestingly, our analysis found an even more pronounced increase in *LmGT3* transcript levels in AMA compared to PRO. This is consistent with results of genetic studies that indicated LmGT3 may have an essential role in the parastiophorous vacuole [52]. Examination of the 13 control genes also found strong agreement (R^2^ = 0.88) between the transcript sizes measured in Northern blots and the transcript lengths established by RNA-seq (S18 Table).

### Enrichment analysis

We tested the differentially expressed genes for enrichment of GO terms, metabolic pathways, Pfam domains, transmembrane domains and signal peptides. We found enrichment in PRO for the GO terms concerning tRNA charging, glycolysis, sterol biosynthesis, central carbon metabolism, respiration (anaerobic) and TCA cycle. In addition we found enrichment in PRO for GO terms plausibly linked to the function of the motile flagellum (microtubule motor activity, dynein complex, microtubule-based flagellum and microtubule based movement) and calcium signalling (calmodulin binding and calcium ion binding) (S19 Table). No GO terms were enriched in the gene set expressed higher in AXA or AMA compared to PRO; this reflects the lack of functional information that is known about these genes. In the gene set expressed higher in AXA than AMA, GO terms associated with proteolytic activity, DNA binding and nucleosomes were enriched (S19 Table), the latter possibly reflecting the higher rate of cell proliferation in AXA.

Analysis for Pfam domain enrichment showed that in PRO, the enriched Pfam domains, like the GO terms, point to functions of the motile flagellum (S20 Table). Both amastigote forms were characterised by higher expression of amastin genes compared to PRO, consistent with the known stage-specificity of a subset of amastin genes [11,53]. Predicted transmembrane domain-containing proteins were significantly enriched overall in both AMA (p = 4.29x10^-10^) and AXA (p = 1.13x10^-16^) compared to PRO. Taken together the enrichment analysis indicates that transcripts with higher expression in PRO include those linked to the function of the motile flagellum, while the gene set with higher expression in early amastigote forms points to a change in surface proteome during differentiation.

Analysis of the presence and absence of the differentially regulated genes in other sequenced trypanosomatid genomes revealed that genes upregulated in the mammalian host were more often found only in *L*. *mexicana* (and other *Leishmania* spp.) than genes that were upregulated in the insect vector (S21 Table). For example, the 1,979 genes that are upregulated in PRO compared to AMA can be clustered into 1,837 orthogroups, of these 1,754 (95%) are found in at least one other *Leishmania* sp., 1,413 (77%) are found in at least one other *Phytomonas* sp. and 1,590 (87%) are found in at least one other *Trypanosoma* sp. genome. In contrast, the 1,853 genes that are upregulated in AMA compared to PRO are clustered into 1,718 orthogroups of which 1,448 (84%) are found in at least one other *Leishmania* sp., 1,019 (59%) are found in at least one other *Phytomonas* sp. and 1,183 (69%) are found in at least one other *Trypanosoma* sp. genome.

### *L*. *mexicana* chromosome 30 is enriched in AMA-upregulated genes

**Fig 8:**
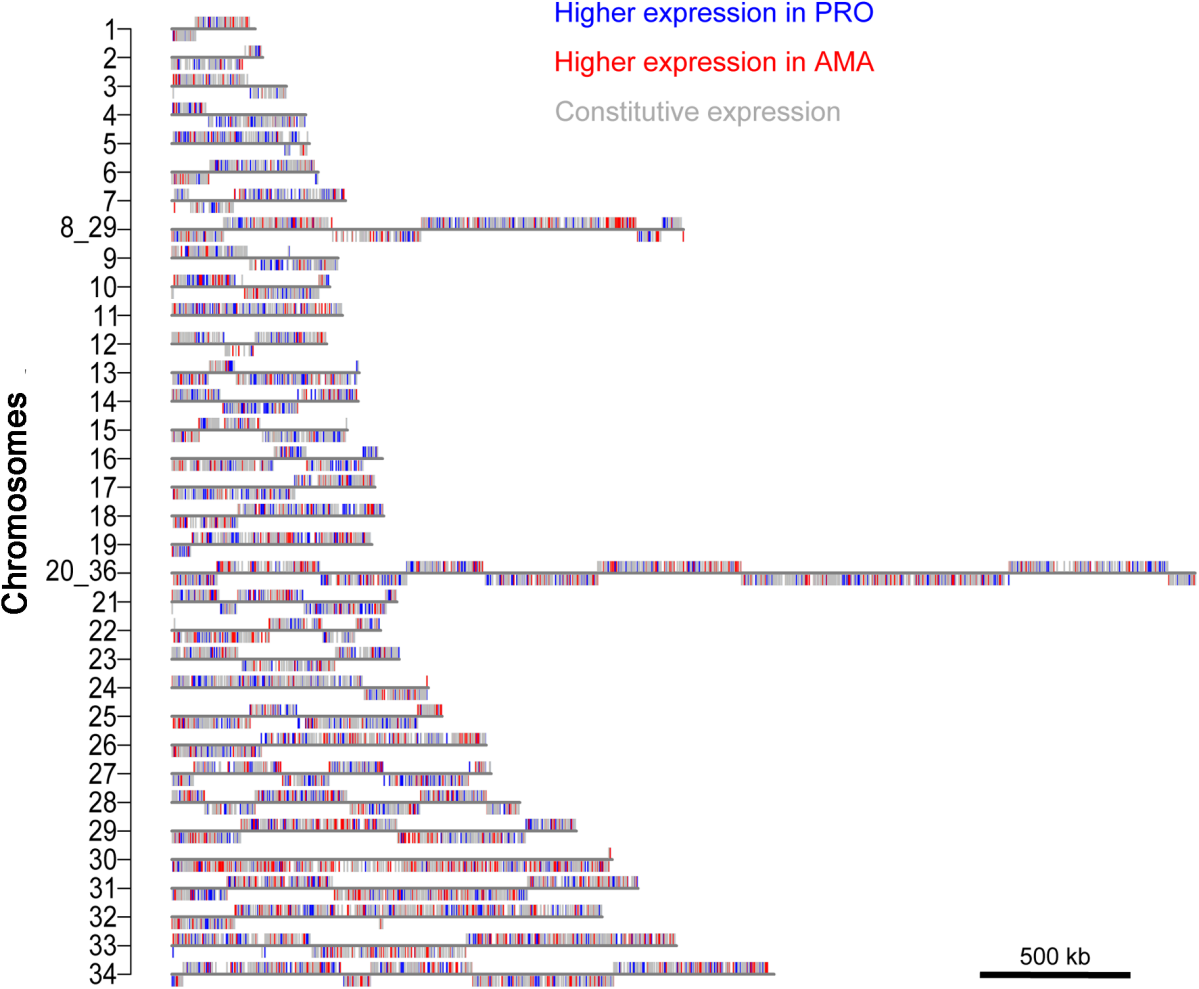
Distribution of differentially expressed genes across chromosomes. Maps of the 34 *L*. *mexicana* chromosomes show the location of genes that are preferentially expressed in AMA (red), PRO (blue) or constitutively expressed (grey).

Genes that are differentially expressed between PRO and AMA were found across all chromosomes (Fig 8) but deviations from the expected numbers (p≤0.05, χ^2^-test) were found for ten chromosomes (S22 Table). Genes upregulated in PRO were over-represented on chromosomes 5, 14 and 21 and underrepresented on chromosomes 08_29 and 30. Genes upregulated in AMA were overrepresented on chromosomes 29, 30 and 33 and underrepresented on chromosomes 4, 14 and 15. The deviation from the expected numbers of transcripts upregulated in AMA was most striking for chromosome 30 (p = 4.03x10^-09^, χ^2^-test) and this was not explained simply by the presence of an amastin gene array on chromosome 30: removal of all amastin genes (defined as genes with a Pfam domain PF07344) from the analysis still showed a significant enrichment of AMA-upregulated genes on chromosome 30 (p = 1.46x10^-06^, χ^2^-test) (S22 Table). A ≥2-fold transcript enrichment in AMA compared to PRO was found for 79 (19%) genes on chromosome 30, of which 20 are novel transcripts, 15 are amastins and the remaining genes encode several amino acid- and other transporters and hypothetical proteins. *L*. *mexicana* chromosome 30 is the homologue of chromosome 31 in other *Leishmania* spp., which has been shown to be supernumerary (typically tetraploid) in all examined *Leishmania* spp. and isolates [7,8]. Our finding that amastigote-upregulated genes are over-represented on this chromosome, together with the independent duplication event of homologous sequences in *T*. *brucei* [54] strongly links the duplication of this chromosome to the adaptation to vertebrate hosts.

## Discussion

This study analysed the transcriptomes of promastigote and early amastigote forms of *L*. *mexicana* to refine gene models and compare transcript abundances in a parasite strain and under culture conditions widely used in studies of *Leishmania* biology. The single nucleotide resolution of the RNA-sequencing data allowed for the first time mapping of *L*. *mexicana* SLAS and PAS on a genome-wide scale and thereby definition of processed transcript boundaries. This allowed a fresh interrogation of gene models and led to the prediction of 9,169 potentially protein coding genes, of which 936 have not been previously described.

The majority of these novel transcripts contain open reading frames that are shorter than the CDS of genes in the current genome annotation. Short open reading frames (sORFs, <100 codons), including those upstream of recognised CDS (uORFs), and peptides encoded by sORFs have attracted a lot of interest since evidence has accumulated in many species from bacteria to humans (reviewed in [55,56], and recently *T*. *brucei* [49]), that some have important functions. Examples range from regulation of protein expression [57,58] to signal transduction within and between cells [59,60] and development [61]. Differentiation of functional sORFs from spurious ORFs that occur in a genome by chance is difficult [62] and we cannot rule out the possibility that some novel transcripts represent trans-spliced and poly-adenylated non-coding transcripts [63] or possibly intermediate products of pre-mRNA processing. Whilst we do not expect that all of these novel ORFs encode proteins, integration of transcriptomic evidence with comparative sequence analysis, protein feature predictions and experimental evidence increases confidence in the prediction of *bona fide* short CDS. In our study, direct MS evidence for 47 of the predicted novel proteins and detection of Pfam-A and-B domains in another 53 sequences provided strong evidence that at least 100 of the novel *L*. *mexicana* transcripts are protein-coding. This is likely to be an underestimation of the true number of novel proteins because the small size of the peptides biases against their detection by conventional protein sample preparation and MS [64,65]. Reciprocal best tblastx analysis of trypanosomatid genomes uncovered a high degree of conservation of the derived amino acid sequences of the novel transcripts. Our results converge with other recent studies [42,49] on a small set of novel transcripts that are widely conserved across trypanosomatids and revealed a larger set of several hundred novel transcript sequences specific to *Leishmania* spp. Small proteins are now recognised to play diverse and important roles, acting predominantly as regulators of diverse cellular processes [66]. 126 novel transcripts are ≥2-fold more abundant in AMA compared to PRO and future studies of their function should consider the possibility that amastigote-derived peptides may have targets in the host cell.

At the level of annotation, we identified two potential causes for mis-annotations of novel transcripts: first, overlap with non-coding RNA loci decreases the confidence three novel genes adjacent to annotated tRNA loci and 18 novel genes that are syntenic with snoRNA loci in *L*. *major*. Few non-coding RNA genes are currently annotated in the *L*. *mexicana* genome and detailed mapping of those was outside the scope of this study but future refinements of the *L*. *mexicana* genome annotation will clarify the status of some of these novel transcripts. Second, 41 novel transcripts mapped to regions of the genome which contain assembly gaps or are known to be incorrectly assembled, including the *PFR2* locus, and should be viewed with caution. Together these make up a small proportion of the 936 novel transcripts and overall we conclude that a substantial proportion of novel transcripts represent a previously undiscovered fraction of the *L*. *mexicana* transcriptome, that may have important, potentially *Leishmania*-specific functions.

The amastigote form of *Leishmania* spp. remains a relatively poorly understood cell and its intracellular lifestyle complicates laboratory studies of its biology. Isolation of amastigotes before RNA extraction could alter gene expression profiles properties, as demonstrated for example for DNA polymerase β transcripts [67]. The dual-RNA sequencing approach allowed us to establish the first global gene expression profile of undisturbed intracellular amastigotes at an early time point after differentiation. Differential expression testing showed a statistically significant difference in abundance (*p* ≤ 0.05) between PRO and AMA for 41% of transcripts, with 14% of transcripts showing a ≥ 2-fold difference. It remains a disputed question to what extent axenically differentiated amastigotes can serve as useful models for amastigote biology. There was a significant difference in abundance in 13% of transcripts between AMA and AXA, but very few showed a ≥ 2-fold difference (only 0.7% of all genes). Thus on a global scale, the transcriptomes of recently differentiated AXA and AMA were much more similar to each other than either was to the promastigote form. Importantly, consultation of published studies of well-characterised stage-regulated transcripts showed good agreement of our RNA-seq results with published Northern blot or qPCR data of intracellular or axenic amastigotes, validating our data. It will be interesting to compare the gene expression patterns of the early amastigotes analysed here with those of amastigotes from infected animal tissues at different time points in an infection.

While we observed roughly equal numbers of differentially regulated genes between PRO and AMA the presence/absence of those genes in other kinetoplastid genomes was markedly different. Specifically, more of the genes that were upregulated in the insect adapted life cycle stage were detectable in other trypanosomatid genomes than those that were upregulated in the mammalian adapted life cycle stage. This raises the question as to the evolutionary history of these genes, i.e. were they invented in the ancestor or *Leishmania mexicana* after its divergence from other lineages (such as *Phytomonas* and *Trypanosoma*) or were they lost from other lineages following divergence from the last common ancestor. Additional genome resources across the breadth of the kinetoplastid tree will help resolve this question.

The gene models reported here enable genome-wide searches for sequence elements contributing to stage-regulation of gene expression. Whilst post-transcriptional control of gene expression in kinetoplastids operates at multiple levels [68] and RNA abundance shows limited correlation with protein levels globally [28,29], our RNA-seq analysis, consistent with many earlier transcriptomic studies, identified genes where stage-regulation of the transcripts correlates strongly with the expression pattern of the protein. These include genes encoding flagellar proteins and surface proteins, both of which have provided paradigms for control of mRNA levels by *cis*-acting elements such as the regulatory elements in the UTRs of PFR2 and the major surface proteins of amastigotes, the amastins [33,69,70]. Further investigation into functionally related cohorts of transcripts might prove fruitful not only for the discovery of additional features and sequence elements regulating their transcript abundances but also master regulatory factors acting on these elements, controlling surface proteome composition or flagellum formation during differentiation.

Whilst alternative uses of SLAS could provide mechanisms for stage-regulation of gene expression, we found that globally, 90% of genes shared the same major SLAS between PRO and AMA, indicating that despite the heterogeneity of sites, a dominant site is used for most genes in both stages. Few of the mapped sites were exclusive to one cell type but differential use of a dominant site in nearly 10% of genes warrants further investigation since alternative trans-splicing may affect the expression or localisation of the protein product, as suggested by recent analyses of differential splicing between bloodstream and procyclic forms of *T*. *brucei* [71,72]. The small numbers of minor SLAS precluded a rigorous *in silico* analysis of their differential usage, a limitation akin to that reported in another comparison between *T*. *brucei* life cycle stages [47].

The organisation of functionally related genes into polycistronic transcription units (PCU) could add another level of control over stage-specificity. Siegel *et al*. [47] analysed this for *T*. *brucei* and found no evidence for co-regulation of genes within a given PCU [47]. However some evidence suggests that the order of genes in their PCU is important for their expression during the cell-division cycle [73]. Transcriptional start sites in *L*. *mexicana* have not yet been mapped but once their locations become known our RNA-seq data of promastigotes and amastigotes can be mapped onto PCUs to test this idea in *Leishmania*.

*Leishmania* spp. are remarkable for their plasticity in chromosome copy number. Amplification of a given chromosome will increase the gene copy number of all genes on that chromosome and, assuming gene dosage affects the level of gene expression, one might expect to find functionally linked genes in *Leishmania* spp. clustered by chromosomes. Our data shows that the distribution of genes up- or downregulated in PRO or AMA diverges significantly from the genome average on ten chromosomes. Chromosome 30 showed the most striking enrichment of AMA-upregulated transcripts, distributed over the entire chromosome. Interestingly, the syntenic block that constitutes *L*. *mexicana* chromosome 30 was duplicated in the *T*. *brucei* clade to form parts of chromosomes 4 and 8, providing opportunities for evolutionary innovations through divergence of paralogous sequences. The 47% of duplicated genes that were retained as paralogous loci showed an enrichment of genes containing TMDs or SPs, suggestive of a function at the host-parasite interface [54]. Independent duplication of this region in *Leishmania* spp. may have assisted adaptation to vertebrate parasitism in both these sister lineages. Our data supports this because of the functionally annotated transcripts that were at least two-fold upregulated in AMA, several are plausibly advantageous to survival in the mammalian host cell, including amino acid transporters (LmxM.30.0330, LmxM.30.0571, LmxM.30.0870, LmxM.30.1820), tryparedoxin (LmxM.30.1960) for detoxification of reactive oxygen species, and one member of the ABC transporter superfamily (LmxM.30.1290)). Aquaglyceroporin 1 (AQP1) (LmxM.30.0020) transports solutes and protects amastigotes from hypoosmotic shock [74]. Advantages of high AQP1 expression in amastigotes are however counterbalanced by the facilitated influx of antimonials, linking higher expression of the gene to greater drug-sensitivity [74,75]. The majority of the upregulated transcripts (including 20 novel transcripts) from chromosome 30 have no known function and focused experiments are now required to discover their importance in amastigote biology.

Attributing functions to these hypothetical proteins is a key challenge for the future and may well identify as yet completely uncharacterised aspects of amastigote biology and virulence and shed more light on the evolution of parasitism and adaptations to specific niches in the host. About half of the genes in the *L*. *mexicana* genome encode hypothetical proteins and our study shows that proteins of unknown function (including putative novel small proteins) dominate among the genes that are most upregulated in amastigotes, not only on chromosome 30 but genome-wide. The ability to perform large-scale unbiased loss of function screens would facilitate the identification of essential genes and those required specifically for survival in the macrophage. Development of such technologies should be a priority to utilise the data generated by this study to its fullest potential.

## Materials and Methods

### Cell culture

Promastigote-form *L*. *mexicana* (WHO strain MNYC/BZ/62/M379) were grown at 28°C in M199 medium (Life Technologies) supplemented with 2.2 g/L NaHCO_3_, 0.005% haemin, 40 mM 4-(2-Hydroxyethyl)piperazine-1-ethanesulfonic acid (HEPES) pH 7.4 and 10% FCS. Axenic amastigotes were generated by dilution of stationary phase promastigotes into Schneider’s Drosophila medium at pH 5.5 [20] to a density of 3x10^6^ cells/ml and incubation at 34°C.

Murine bone marrow cells were harvested as described in [18]. *In vitro* maturation in 20% L929 conditioned medium resulted in cells expressing the murine macrophage markers F4/80 [76] and MAC-1 [77], whilst being negative for the granulocyte marker GR-1 as assessed by flow-cytometry.

To generate intracellular and axenic amastigotes, promastigotes were left to grow into stationary phase (from 1x10^5^ to 2x10^7^ cells/ml) and then incubated with BMDMs at a parasite to macrophage ratio of 20 for 2h before washing off remaining extracellular parasites. 24h post infection, glass coverslips placed in the culture dish were fixed with methanol and stained with propidium iodide and DAPI [78] to count the proportion of infected macrophages and number of intracellular amastigotes.

### RNA extraction and quality control

*Leishmania*-infected BMDMs were harvested 24h post-infection and axenic amastigotes were harvested 24h post-transfer to differentiation medium. Promastigotes were harvested in late exponential growth phase (around 1x10^7^ cells/ml). RNA was extracted using a QIAgen RNeasy Mini Kit as per manufacturer’s instructions. Samples AMA, AXA and PRO 2‐3 were subsequently treated using Ambion Turbo DNAase kit. RNA purity and integrity was assessed by OD 260/280 and OD 260/230 measurements and visual inspection of electropherograms produced on a Bioanalyzer2100 (Agilent Technologies),

### RNA sequencing

The cDNA libraries were prepared and sequenced at the Beijing Genomics Institute (Shenzhen, China). In brief, polyadenylated RNA was purified from total RNA, converted to cDNA using random hexamer primers sheared and size selected for fragments ~200bp in length using the Illumina TruSeq RNA Sample Preparation Kit v2. RNAseq of the resulting “Library 1” was used for both mapping of splice acceptor sites and determination of transcript abundances. For determination of polyadenylation addition sites, two replicate samples for each cell type were used to generate a second cDNA library (Library 2). The protocol was the same however instead of using random hexamers for cDNA synthesis a 5’-T15VN-3’ oligonucleotide (V = A, G or C; N = T, A, G or C) [42] was used to enrich for 3’ ends of transcripts. Sequencing was performed on an Illumina Hiseq 2000 (Illumina, CA) platform.

### Identification of trans-splice and polyadenylation acceptor sites

The paired end reads generated from both cDNA libraries were processed as described in [46]. Briefly, reads from Library 1 containing the final 12 bases of the spliced leader sequence (i.e. TGTACTTTATTG) were extracted, spliced-leader excised, and the remaining read mapped to the *L*. *mexicana* genome to record the positions of the trans-splice sites (SLAS). Reads that were less than 21 nucleotides in length after extraction of the spliced-leader sequence were discarded. Reads from Library 2 containing 5 or more A nucleotides at end of a read (or 5 or more T at the start) were identified, A or T tails removed from the read and the remainder and mapped to the genome. PAS were recorded if the genomic locus itself contained no equivalent run of As or Ts at the mapped position. Sites detected fewer than 9 times across the 9 samples (SLAS) or 6 times across the 6 samples (PAS) were discarded.

### RNA-seq guided annotation of predicted CDS and assignment of SLAS and PAS to CDS

*SLAS* ***‐*** *based gene predictions*. Reads from cDNA Library 1 were initially mapped to version 4.1 of the *L*. *mexicana* genome and the positions of the trans-splice sites (SLAS) were recorded. The sequence between each SLAS and the next downstream SLAS on the same strand was scanned for ATG trinucleotides (possible translation start codons), and TAA, TAG and TGA trinucleotides (possible translation stop codons). From each detected ATG the downstream sequence was scanned for in‐frame stop codons. Where the distance between the ATG and the first in‐frame stop codons was >75 nt it was recorded in GFF format as a predicted ORF of ≥ 25 codons (pORF). If no pORF was detected between a SLAS, and the next downstream SLAS, the search was re-initiated from the downstream SLAS. Where a pORF was found in the same “inter‐SLAS” space as an annotated CDS, we compared the co-resident ORFs. (1) If the pORFs and the annotated CDS shared the same stop but had a different start codon, these were divided into ‘extensions’ and ‘truncations’ depending on whether the pORF start codon was upstream or downstream of the reference start codon. All extensions were recorded; truncations were manually curated, taking into account the SLAS‐pattern and frequencies (truncated CDS were only accepted when consistent with the dominant cluster of splice sites or a unique splice site), read‐coverage and reference to existing annotations and conservation between kinetoplastid species using information on TriTrypDB. In ambiguous cases, the pORF was discarded in favour of the reference CDS. (2) When a pORF was found in the same “inter‐SLAS” space as an entire annotated CDS but with a different stop codon, it was recorded as a putative uORF. All recorded CDS were compiled into GFF format and compared to the reference annotation, to fill in annotated CDS that were not captured by the SLAS‐based CDS annotation.

All identified SLAS and PAS were then assigned to individual recorded CDS using SLaP Mapper [46]. The resulting PAS positions were used to filter out likely false positives from the detection of novel CDS. All novel CDS without PAS were removed and all SLAS and PAS were reassigned using SLaP Mapper. Subsequently, all novel CDS with more than 10% of PAS internal to the CDS were excluded; all remaining CDS with internal PAS were manually inspected and candidates with even distribution of PAS over the entire CDS were removed. All SLAS and PAS were again reassigned to remaining CDS. Finally, where a novel CDS was recorded downstream of a reference CDS without an assigned PAS, sequencing read‐coverage was considered to exclude ‘novel’ CDS that lay within the likely 3’UTR of the reference CDS. SLAS and PAS were then re‐assigned to the remaining 9,169 CDS and transcript models were generated for each CDS from the 5’ distal SLAS to the 3’ distal PAS. The Integrative Genome Browser (IGV) [79,80] was used for visualisation.

### Proteomic analysis of promastigote and axenic amastigote lysates

To prepare whole cell protein lysates, PRO were washed three times in PBS and AXA were washed three times in PBS with protease inhibitors (50 μM Leupeptin hydrochloride, 5μM E-64). Cells were lysed in ice-cold lysis buffer (8M Urea in 125mM Tris, pH 6.8 with 1% Sodiumdeoxycholate and protease inhibitors as above). Lysis was performed on ice with 5 s vortexing every 30 s for 5 min. Protein concentration was determined using Thermo Scientific Pierce 660nm Protein Assay as per manufacturer’s instructions. For mass-spectrometric analysis of AXA and PRO protein samples, detergent was removed by precipitation with 0.5% (v/v) trifluoroacetic acid and centrifugation at 13,000 g for 10 min. Proteins contained in the supernatant were denatured in 8M Urea, 10 mM dithiothreitol and 10mM iodoacetamide. In-solution tryptic digests were performed at 10-fold excess of trypsin. Samples were desalted on a C18-column and injected into an HPLC-coupled QExactive mass-spectrometer. In addition, promastigote cells were partitioned into multiple discrete fractions to facilitate detection of low abundance peptides. Here cells were manually disrupted, separated by sucrose gradient and individual sucrose fractions subjected to solubilisation in 1% octylglycoside to separate fractions into soluble and insoluble pools prior to analysis by mass spectrometry. All resulting.mgf files were combined and spectra were searched against custom data-bases in the Central Proteomics Facility Pipeline [81] allowing for two missed tryptic cleavages with a precursor tolerance of 20 ppm, fragment tolerance of 0.1 Da with fixed Carbamidomethyl and variable N-terminal acetylation and Oxidation (M). Quantitation tolerance was set to 0.02.

The raw mass-spectrometry data from the study by Paape *et al*. [82] was converted to.mgf files and searched by the Central Proteomic Facility Pipeline at the Sir William Dunn School of Pathology [81]. Search parameters were as described above, except that the Fragment Tolerance was increased to 0.5 Da. All peptide-data was exported from MASCOT [83] and label free quantitation was performed according to [84].

### Reciprocal best blast analysis

Reciprocal Best Blast (RBB) [85] analyses were performed using BlastAll software run within a wrapping R script. In the initial query the novel transcript sequences were searched against the target genome. The sequence of the highest ranking hit, was extracted and used in a search against all transcript sequences of *L*. *mexicana*. When the initial query sequence was the highest-ranking return hit, a RBB hit was detected and the-log10 (e‐value) of the return hit recorded. A matrix of all RBB results was generated using a custom script and plotted using the heatmap() function from the “stats” library in R, permitting for hierarchical clustering of transcripts based on conservation pattern using hclust() defaults, but retaining a manually determined order of genomes, based on the evolutionary relationship of kinetoplastids [86]. Genome sequences for *L*. *major* Friedlin, *L*. *donovani* BPK282A1, *L*. *infantum* JPCM5, *L*. *braziliensis* MHOM/BR/75/M2904, *L*. *panamensis* MHOM/COL/81/L13, *L*. *tarentolae* Parrot-TarII, *C*. *fasciculata* Cf-Cl, *E*. *monterogeii* LV88, *T*. *brucei* TREU927, *T*. *congolense* IL3000 and *T*. *cruzi* CL Brener‐Esmeraldo‐like were obtained from TriTrypDB v.7.0. Phytomonas EM1 and HART1 genomes are from [87].

### Transcript profiling and DESeq testing

Sequence data was quality-trimmed using Trimmomatic [88] and aligned to a hybrid *L*. *mexicana*::*Mus musculus* genome with Bowtie 2 and transcript abundances estimated using RSEM [89]. The gene models for *L*. *mexicana* were as defined from our data and Mus_musculus.GRCm38.75.cdna.all.fa from Ensemble (www.ensembl.org). To compensate for unequal library sizes due to the presence or absence of mouse RNA, effective counts corresponding to *Leishmania* transcripts were isolated and counts were median normalized using the default method as used by DESeq2 [90] followed by differential expression analysis using DEseq2. Enrichment analyses were done with GOseq [91].

### Inference of orthologous sequence groups

The predicted protein sequences for a representative set of kinetoplastid species were obtained online from TriTrypDB. These comprised *Phytomonas EM1*, *Phytomonas HART1*, *Phytomonas serpens*, *Trypanosoma brucei TREU927*, *Trypanosoma congolense*, *Trypanosoma cruzi*, *Trypanosoma grayi*, *Trypanosoma rangeli*, *Trypanosoma vivax*, *Leishmania braziliensis*, *Leishmania donovani*, *Leishmania infantum*, *Leishmania major*, and *Leishmania tarentolae*. These proteins were subject to orthologous gene group inference using OrthoFinder [92] using the default program settings.

### Ethics statement

Carcasses for the extraction of bone marrow cells were supplied by Oxford University Biomedical Services. The animals were not killed specifically for our use, and as they were killed by a Schedule 1 method (as per the Animals (Scientific Procedures) Act 1986) licence authority was not required.

### Accession numbers

The sequencing data was deposited in the ArrayExpress repository, accession E-MTAB-3312.

## Supporting Information

S1 Fig Quantification of parasite load in infected macrophages. (A) Top panel: phase contrast image of BMDM population. Bottom panel: fluorescence image showing stained DNA of BMDMs (arrowheads point to two examples of BMDM nuclei) and intracellular *L*. *mexicana* (arrows indicate two BMDMs infected with multiple parasites). (B) Parasite load in infected macrophages determined from examination of cell populations as shown in (A).
(TIF)

S2 Fig Quantification of *L*. *mexicana* rRNA in mixed RNA samples from infected BMDMs. Total RNA from *L*. *mexicana*, uninfected and infected BMDMs was analysed on an Agilent 2100 Bioanalyzer. The resulting electropherograms show the different rRNA peaks in the two species. *L*. *mexicana* shows a peak for the SSU rRNA and the LSU rRNA is fragmented into two large molecules (LSU α and LSU β) and four small ones. Mouse rRNA appears as two peaks, 18S and 28S. Distinct peaks for leishmanial and murine rRNA can be distinguished in the infected BMDM RNA (AMA1 sample shown as example). The ratio of the LSU β (red arrow) to 28S peak (blue arrow) was used to determine the relative amount of leishmanial rRNA in the mixed samples.
(TIF)

S3 Fig Analysis of correlation between UTR lengths and expression levels. (A) Correlation between 5’ and 3’ UTR length in nucleotides (nt) on the same gene. (B) Correlation between expression levels and length of 5’ UTR. (C) Correlation between expression levels and length of 3’ UTR.
(TIF)

S4 Fig FPKM distribution. (A) Histograms showing the distribution of FPKM values in all nine samples. For AMA1-3 only FPKM values of transcripts mapped to the *L*. *mexicana* genome are shown. Numbers in brackets indicate mean/median FPKM values, respectively. (B) Coefficient of variation for measured genes, showing the mean, interquartile range and full data range; binned according to the expression level of the gene.
(TIF)

S1 Table List of mapped SLAS. GFF feature record Columns are <seqname>, <source>, <feature>, <start>, <end>, <score> (“.” denotes no score), <strand> (“.” denotes not relevant), <frame>, [attribute]
(XLSX)

S2 Table List of mapped PAS. GFF feature record Columns are <seqname>, <source>, <feature>, <start>, <end>, <score> (“.” denotes no score), <strand> (“.” denotes not relevant), <frame>, [attribute]
(XLSX)

S3 Table List of extended CDS predictions. GFF feature record Columns are <seqname>, <source>, <feature>, <start>, <end>, <score> (“.” denotes no score), <strand> (“.” denotes not relevant), <frame>, [attribute]
(XLS)

S4 Table List of truncated CDS predictions. GFF feature recordColumns are <seqname>, <source>, <feature>, <start>, <end>, <score> (“.” denotes no score), <strand> (“.” denotes not relevant), <frame>, [attribute]
(XLS)

S5 Table List of novel CDS predictions. GFF feature record Columns are <seqname>, <source>, <feature>, <start>, <end>, <score> (“.” denotes no score), <strand> (“.” denotes not relevant), <frame>, [attribute] Every novel transcripts was given a unique IDs in the format LmxM.[number of chromosome]_[position of last nucleotide of stop codon of predicted CDS], for example: LmxM.01_107651.
(XLSX)

S6 Table Reciprocal best tblastx analysis of conserved and novel genes. (XLSX)

S7 Table Mass-spectrometric evidence for novel proteins obtained from proteomic analysis of AXA and PRO. (XLSX)

S8 Table Mass-spectrometric evidence for novel proteins obtained from proteomic analysis of intracellular amastigotes. (XLSX)

S9 Table Identification of Pfam domains in predicted novel proteins. (XLSX)

S10 Table Mass spectrometry evidence for extended CDS. (XLSX)

S11 Table List of uORFs. GFF feature record Columns are <seqname>, <source>, <feature>, <start>, <end>, <score> (“.” denotes no score), <strand> (“.” denotes not relevant), <frame>, [attribute]
(XLS)

S12 Table Fragments per kilobase of transcript per million mapped reads (FPKM) for each gene. (XLSX)

S13 Table Pearson correlation coefficients. (XLSX)

S14 Table List of genes in the top percentile of FPKM for AMA, AXA and PRO. (XLSX)

S15 Table Differential expression analysis PRO vs. AMA. (XLSX)

S16 Table Differential expression analysis PRO vs. AXA. (XLSX)

S17 Table Differential expression analysis AXA vs. AMA. (XLSX)

S18 Table Comparison of RNA-seq data with published northern blot data for *L*. *mexicana* transcripts. (DOCX)

S19 Table GO term and pathway enrichment summary. (XLSX)

S20 Table Pfam-A and Pfam-B enrichment summary. (XLSX)

S21 Table Orthogroup analysis. (XLSX)

S22 Table Distribution of differentially expressed genes across *L*. *mexicana* chromosomes. (XLSX)
