# Supplementary figures and images for: Comparative Life Cycle Transcriptomics Revises Leishmania mexicana Genome Annotation and Links a Chromosome Duplication with Parasitism of Vertebrates
Source: PLoS Pathog. 2015 Oct 9;11(10):e1005186. doi: 10.1371/journal.ppat.1005186 (PMC4599935; doi:10.1371/journal.ppat.1005186)

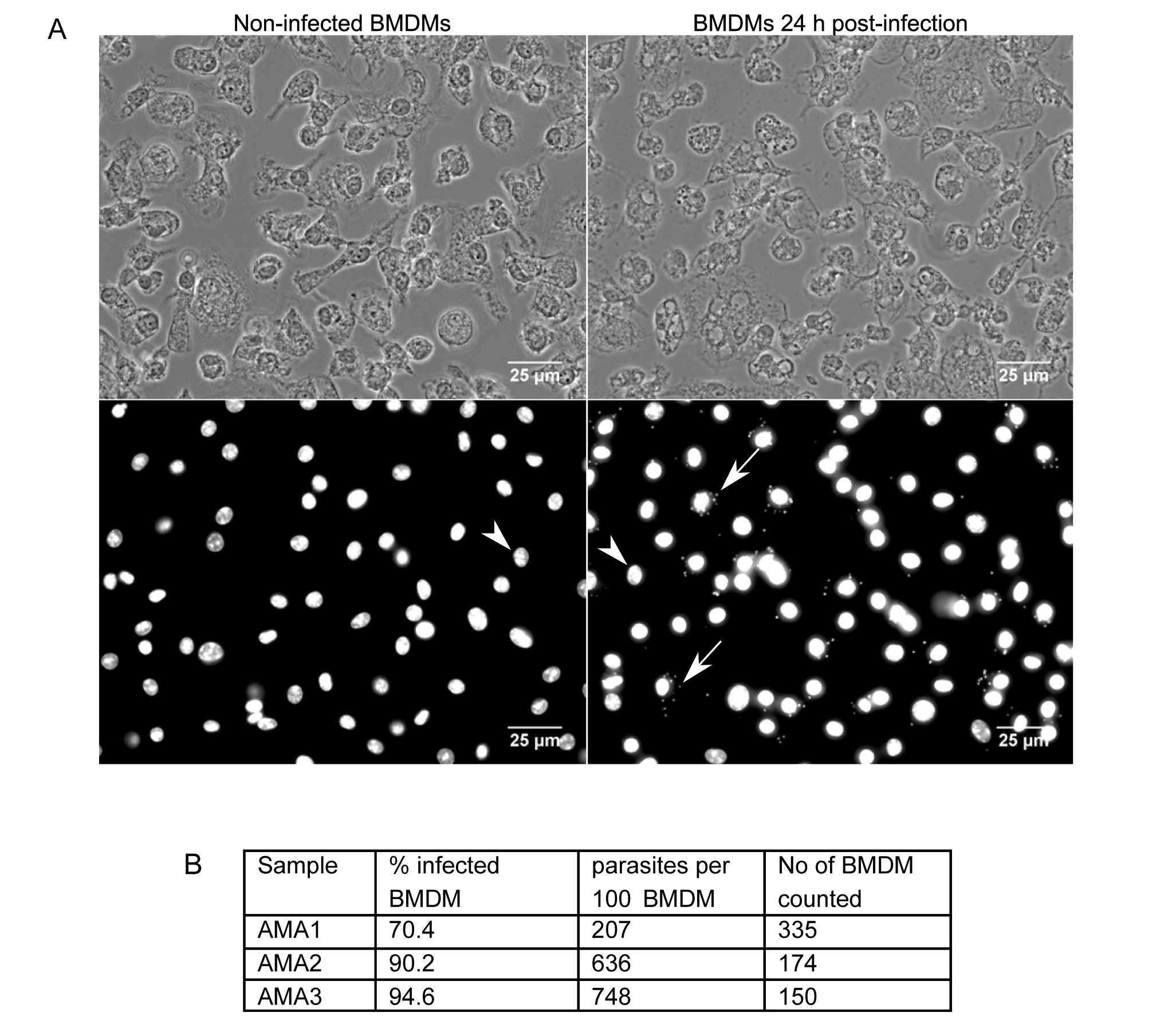

Supplement: S1 Fig — (A) Top panel: phase contrast image of BMDM population. Bottom panel: fluorescence image showing stained DNA of BMDMs (arrowheads point to two examples of BMDM nuclei) and intracellular L. mexicana (arrows indicate two BMDMs infected with multiple parasites). (B) Parasite load in infected macrophages determined from examination of cell populations as shown in (A). (TIF) [file ppat.1005186.s001.tif]

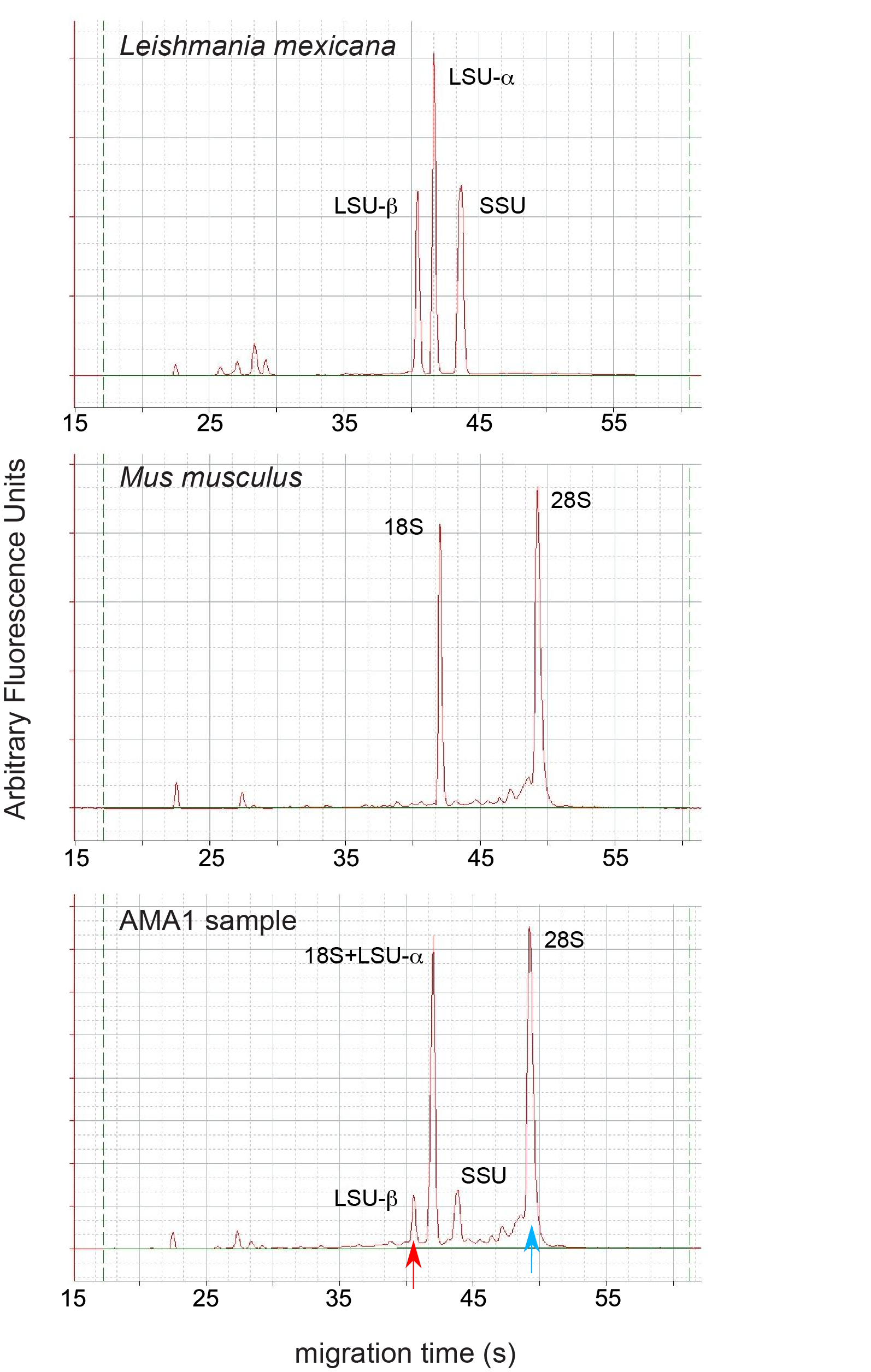

Supplement: S2 Fig — Total RNA from L. mexicana, uninfected and infected BMDMs was analysed on an Agilent 2100 Bioanalyzer. The resulting electropherograms show the different rRNA peaks in the two species. L. mexicana shows a peak for the SSU rRNA and the LSU rRNA is fragmented into two large molecules (LSU α and LSU β) and four small ones. Mouse rRNA appears as two peaks, 18S and 28S. Distinct peaks for leishmanial and murine rRNA can be distinguished in the infected BMDM RNA (AMA1 sample shown as example). The ratio of the LSU β (red arrow) to 28S peak (blue arrow) was used to determine the relative amount of leishmanial rRNA in the mixed samples. (TIF) [file ppat.1005186.s002.tif]

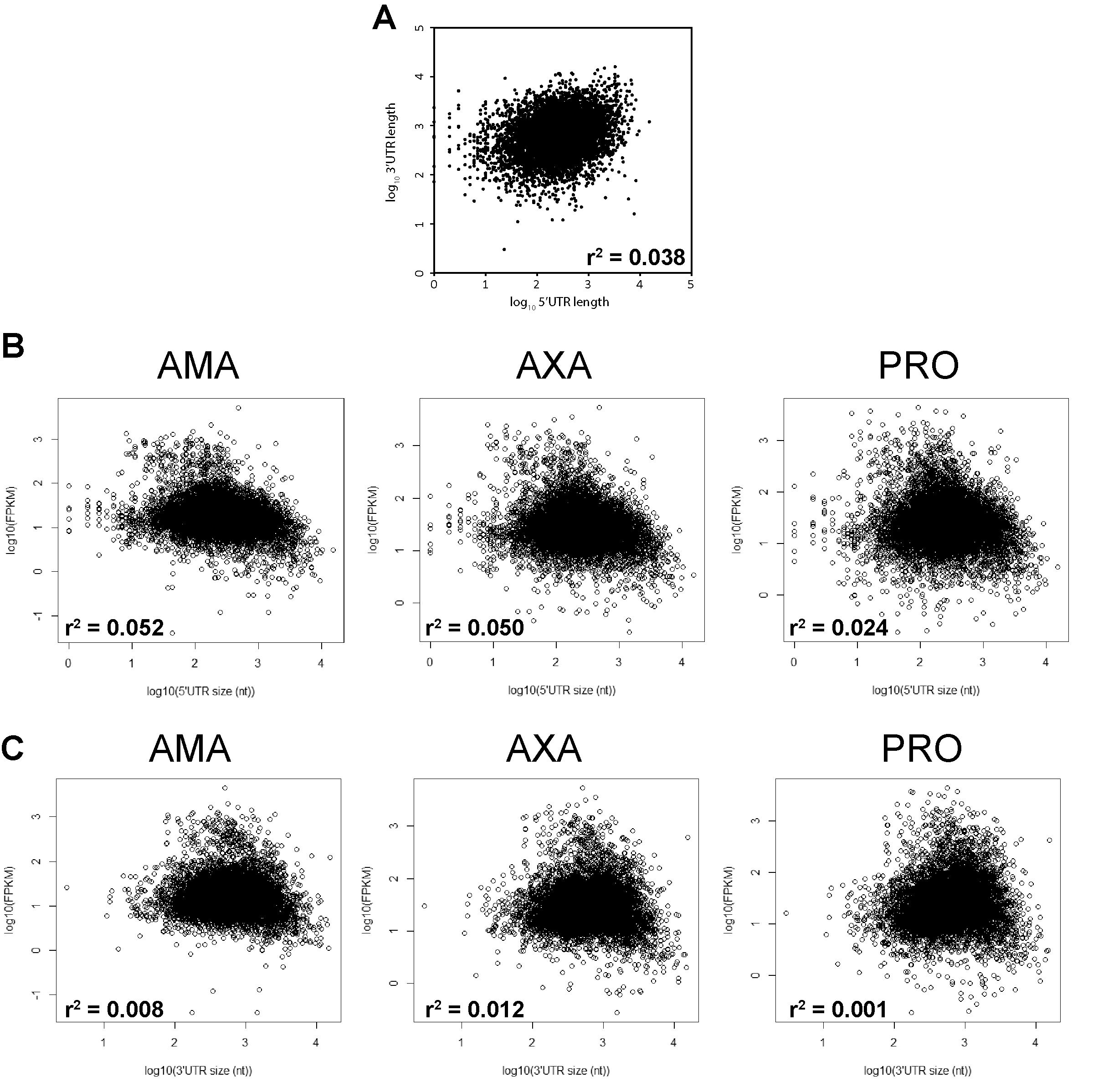

Supplement: S3 Fig — (A) Correlation between 5’ and 3’ UTR length in nucleotides (nt) on the same gene. (B) Correlation between expression levels and length of 5’ UTR. (C) Correlation between expression levels and length of 3’ UTR. (TIF) [file ppat.1005186.s003.tif]

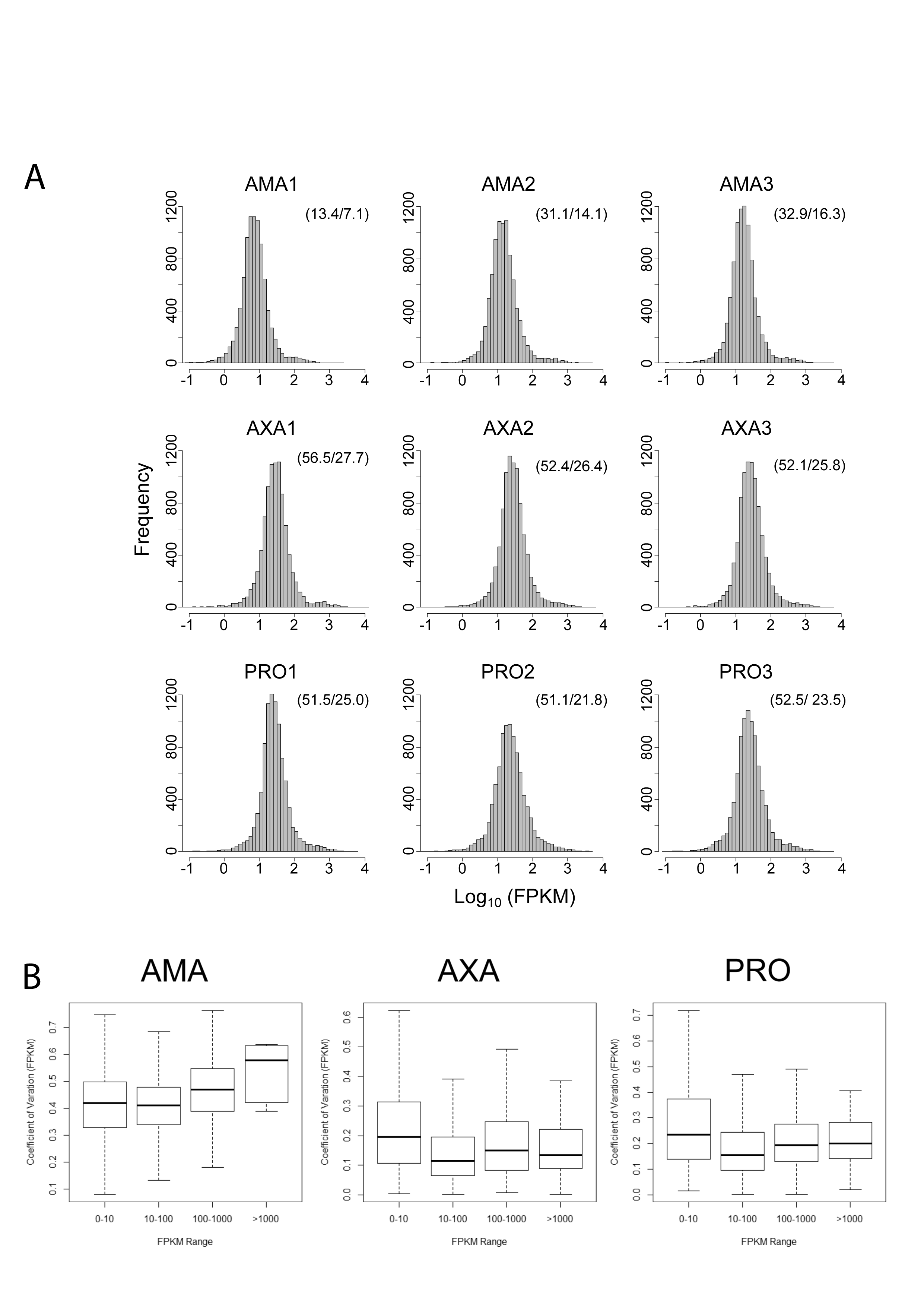

Supplement: S4 Fig — (A) Histograms showing the distribution of FPKM values in all nine samples. For AMA1-3 only FPKM values of transcripts mapped to the L. mexicana genome are shown. Numbers in brackets indicate mean/median FPKM values, respectively. (B) Coefficient of variation for measured genes, showing the mean, interquartile range and full data range; binned according to the expression level of the gene. (TIF) [file ppat.1005186.s004.tif]
