# Supplementary material for: Comparative Life Cycle Transcriptomics Revises Leishmania mexicana Genome Annotation and Links a Chromosome Duplication with Parasitism of Vertebrates
Source: PLoS Pathog. 2015 Oct 9;11(10):e1005186. doi: 10.1371/journal.ppat.1005186 (PMC4599935; doi:10.1371/journal.ppat.1005186)
Supplement: S18 Table — (DOCX) [file ppat.1005186.s022.docx]

**Table S18: Comparison of RNA-seq data with published Northern blot data for *L. mexicana* transcripts.**

| ***L. mexicana* Gene ID** | **Gene product** | **Reference** | **Transcript size (kb)** | | **Result of Northern blot analysis: relative transcript abundances** (a) | **DESeq comparison**  **PRO vs. AMA** | | **DESeq comparison**  **PRO vs. AXA** | |
| --- | --- | --- | --- | --- | --- | --- | --- | --- | --- |
|  |  |  | **Northern blot** | **RNA-seq** |  | **log2 Fold Change** | **padj** | **log2 Fold Change** | **padj** |
| LmxM.16.0390 | VG7A5 | [[1](#_ENREF_1)] | 2.5, 0.3 | 4.1 (b) | AXA > PRO | -0.07 | 8.00E-01 | 0.10 | 7.16E-01 |
| LmxM.16.1410 | "PFR2 downstream gene 2" | [[2](#_ENREF_2)] | 1.8 | 2.1 | PRO > AXA | 0.35 | 1.39E-01 | 0.15 | 6.62E-01 |
| LmxM.16.1420 | "PFR2 downstream gene 1" | [[2](#_ENREF_2)] | 4 | 3.7 | PRO > AXA | 1.95 | 3.18E-15 | 0.50 | 1.04E-01 |
| LmxM.16.1430 | PFR2-A or PFR2-B (c) | [[2](#_ENREF_2)] | 3.1 | 3.2 | PRO > AXA | 2.73 | 1.04E-61 | 2.11 | 2.23E-55 |
| LmxM.16_577896 | "PFR2 upstream gene 1" | [[2](#_ENREF_2)] | 1.8 | 1.8 | PRO = AXA | -0.29 | 2.33E-01 | -0.33 | 2.27E-01 |
| LmxM.16.1440 | "PFR2 upstream gene 2" | [[2](#_ENREF_2)] | 6.2 | 5.9 | PRO / AXA is 0.5 | -0.03 | 9.10E-01 | -0.33 | 1.55E-02 |
| LmxM.21.1860 | A850 (beta-tubulin) | [[3](#_ENREF_3)] | 2.8 | 2.8 | AXA > PRO and AMA > PRO | -2.52 | 2.11E-74 | -1.45 | 2.90E-16 |
| LmxM.23.1060 | HASPB | [[4](#_ENREF_4)] | 2 | 1.9 | AXA > PRO | -2.54 | 3.77E-28 | -1.35 | 2.20E-05 |
| LmxM.30.2310 | 3′-nucleotidase / nuclease | [[5](#_ENREF_5)] | 2.7 | 2.6 | PRO > AMA | 1.80 | 4.59E-18 | 0.64 | 5.74E-02 |
| LmxM.33.3645 | A600-4 | [[3](#_ENREF_3),[6](#_ENREF_6)] | 3.3 | 4.4 | AXA > PRO and AMA > PRO | -5.60 | 2.24E-102 | -3.21 | 3.67E-46 |
| LmxM.36.6280 | LmGT3 | [[7](#_ENREF_7)] | 7.5 | 7 | PRO = AXA | -1.95 | 1.09E-13 | -0.55 | 9.40E-03 |
| LmxM.36.6290 | LmGT2 | [[7](#_ENREF_7)] | 3.5 | 3.3 | PRO > AXA | 2.43 | 5.21E-17 | 1.01 | 2.63E-03 |
| LmxM.36.6300 | LmGT1 | [[7](#_ENREF_7)] | 8 | >6.1 (d) | PRO = AXA | 1.19 | 2.02E-06 | 0.36 | 2.15E-01 |

(a) Only those relevant for the comparison with the RNA-seq data are shown; some of the studies also analysed metacyclic promastigotes.

(b) The CDS for LmxM.16.0390 is 2.9 kb, i.e. larger than the reported Northern blot transcript.

(c) The *PFR2* array is incompletely assembled in the current version of the *L. mexicana* genome. The measured transcript lengths and mapped 3’UTR in Moore et al. [[2](#_ENREF_2)] suggest that LmxM.16.1430 represents the *PFR2-A* and *PFR2-B* gene and the adjacent, partially assembled gene LmxM.16.1425partial is *PFR2-C*.

(d) Due to a gap in the genome assembly, a precise measurement of the *LmGT1* transcript size is currently not possible from RNA-seq data.

References

[1] Liu K, Zinker S, Arguello C, Salgado LM (2000) Isolation and analysis of a new developmentally regulated gene from amastigotes of *Leishmania mexicana mexicana*. Parasitol Res 86: 140-150.

[2] Moore LL, Santrich C, LeBowitz JH (1996) Stage-specific expression of the *Leishmania mexicana* paraflagellar rod protein PFR-2. Mol Biochem Parasitol 80: 125-135.

[3] Bellatin JA, Murray AS, Zhao M, McMaster WR (2002) *Leishmania mexicana*: identification of genes that are preferentially expressed in amastigotes. Exp Parasitol 100: 44-53.

[4] Depledge DP, MacLean LM, Hodgkinson MR, Smith BA, Jackson AP, et al. (2010) *Leishmania*-specific surface antigens show sub-genus sequence variation and immune recognition. PLoS Negl Trop Dis 4: e829.

[5] Sopwith WF, Debrabant A, Yamage M, Dwyer DM, Bates PA (2002) Developmentally regulated expression of a cell surface class I nuclease in *Leishmania mexicana*. Int J Parasitol 32: 449-459.

[6] Murray A, Fu C, Habibi G, McMaster WR (2007) Regions in the 3' untranslated region confer stage-specific expression to the *Leishmania mexicana* a600-4 gene. Mol Biochem Parasitol 153: 125-132.

[7] Burchmore RJ, Landfear SM (1998) Differential regulation of multiple glucose transporter genes in *Leishmania mexicana*. J Biol Chem 273: 29118-29126.
